# Functional Dissection of TP63 Dual-Promoter Regulatory Elements Reveals Context-Dependent Responsiveness to FOXA2, NKX2–1, SOX9, and TP63-Related Inputs

**DOI:** 10.3390/cimb48090882

**Published:** 2026-08-30

**Authors:** Yijian Lin, Xiaoshan Su, Dachun Wang, Yiming Zeng

**Affiliations:** 1 Department of Respiratory and Critical Care Medicine, Second Affiliated Hospital of Fujian Medical University, Quanzhou 362000, China; 2 Fujian Key Laboratory of Lung Stem Cells, Second Affiliated Hospital of Fujian Medical University, Quanzhou 362000, China; 3 The Brown Foundation Institute of Molecular Medicine for the Prevention of Human Diseases, University of Texas Medical School at Houston, Houston, TX 77030, USA; 4 Fujian Clinic Research Center of Interventional Pulmonology, Second Affiliated Hospital of Fujian Medical University, Quanzhou 362000, China; 5 NHC Key Laboratory of Etiological Epidemiology of Chronic Diseases with High Incidence in Fujian-Taiwan Area (Co-Construction), Fujian Medical University, Fuzhou 350122, China

**Keywords:** TP63, alternative promoter, promoter responsiveness, gene regulation, luciferase reporter assay, ChIP-qPCR, A549 cells

## Abstract

The TP63 locus generates TAp63 and ΔNp63 isoforms through alternative promoter usage, but the cis-regulatory features that confer differential promoter responsiveness remain incompletely defined. Here, we functionally dissected distal TAp63-associated and proximal ΔNp63-associated TP63 promoter regions using reporter-based assays in A549 cells. Rabbit TP63 promoter fragments were selected from distal and proximal promoter-associated regions and analyzed by serial deletion and site-directed mutagenesis. Deletion mapping identified discrete promoter intervals that positively or negatively affected reporter activity, while motif perturbation revealed promoter-context-dependent contributions of predicted FOXA1/2, NKX2–1, SOX9, and TP63-related regulatory elements. Endogenous ChIP-qPCR at corresponding human TP63 promoter-associated regions provided local enrichment evidence for selected transcription-factor-associated signals, and FOXA2 or SOX9 depletion shifted endogenous TAp63 and ΔNp63 protein abundance. These findings identify candidate TP63 promoter intervals and transcription-factor-responsive motifs that differentially affect dual-promoter reporter output. The study provides a promoter-level framework for investigating how TP63 promoter responsiveness is influenced by promoter architecture, motif composition, and cellular regulatory background within a heterologous reporter context, while highlighting the need for future validation in more physiological human epithelial systems.

## 1. Introduction

TP63 is a well-established example of alternative promoter regulation. The TP63 locus uses two promoters to generate the N-terminally distinct TAp63 and ΔNp63 isoforms, which differ in transcriptional activity and biological output [1]. In the respiratory epithelium, TP63 is closely linked to progenitor and basal-cell programs: embryonic TP63 positive cells contribute to distinct adult airway stem-cell pools [2,3], and postnatal basal cells marked by TP63 function as stem cells during epithelial maintenance and repair [4,5,6,7]. However, the promoter-level cis-regulatory features that shape differential TP63 promoter responsiveness remain incompletely understood.

Reporter-compatible cellular systems remain useful for dissecting promoter architecture because they allow defined cis-elements, candidate transcription-factor-associated motifs, and reporter constructs to be perturbed in a uniform experimental background. Such systems cannot replace primary, organoid, or developmental models [8,9,10,11,12], but they provide a practical platform for identifying promoter intervals and motifs that merit further mechanistic testing. Therefore, the present study used A549 cells as a transfection-compatible epithelial cancer-derived assay background for TP63 promoter-reporter dissection.

FOXA1, FOXA2, NKX2-1, and SOX9 are plausible upstream regulators of TP63 promoter selection because they belong to gene-regulatory circuits that shape foregut and lung epithelial identity. Foxa1/2 cooperate during lung morphogenesis to control branching, epithelial differentiation, and respiratory epithelial gene expression [9,13,14,15]. Sox2 and Sox9 contribute to anterior foregut patterning and proximal-distal epithelial organization in the developing lung [16,17,18,19], whereas NKX2-1 identifies early lung-lineage progenitors that can be isolated during human pluripotent stem cell differentiation [9,11]. These observations make FOXA1/2, NKX2-1, SOX9, and TP63-related inputs biologically relevant candidates for testing promoter-selective responsiveness at the TP63 locus.

In this study, we examined distal TAp63-associated and proximal ΔNp63-associated TP63 promoter regions using rabbit promoter-reporter constructs, serial deletion, site-directed mutagenesis, endogenous ChIP-qPCR, public expression and chromatin-context analyses, and siRNA-mediated perturbation in A549 cells. Rabbit sequences were used as promoter-template sources for reporter-based exploration because rabbit provides a longer pseudoglandular-stage interval than mouse or rat and is phylogenetically closer to human [20,21,22,23]. The study was designed to identify candidate cis-regulatory segments and transcription-factor-responsive motifs that shape TP63 dual-promoter reporter output, and to generate a promoter-level framework for future validation in more native epithelial systems. Importantly, these rabbit promoter fragments were used as comparative promoter templates for functional dissection and were not intended to represent direct substitutes for endogenous human TP63 promoter regulation.

## 2. Methods

### 2.1. Bioinformatic Analysis of Public RNA-Seq Datasets

To provide transcriptomic context for the A549-based promoter assays, we performed a comparative analysis using public gene-expression datasets deposited in the Gene Expression Omnibus (GEO). The reference set included hiPSC-derived differentiation stages, including undifferentiated hiPSCs, definitive endoderm, anterior foregut endoderm, and NKX2-1-positive lung-directed cells from GSE83310; airway epithelial reference samples from GSE124253 and GSE31449; alveolar samples from GSE120908; and untreated baseline A549 samples from GSE103987 (Supplementary Table S2). Because these datasets were generated by different laboratories, platforms, and processing pipelines, we did not attempt formal batch-corrected integration or infer equivalence between A549 and any primary or developmental epithelial population. Instead, the analysis was used only as a descriptive positioning framework.

For each GEO series, processed expression values available from the GEO record or accompanying Supplementary Files were retrieved. Because these datasets were generated using different laboratories, platforms, and processing pipelines, the expression matrices were analyzed within a descriptive comparative framework and were not interpreted as directly comparable absolute expression measurements. Gene identifiers were harmonized to official human gene symbols where possible, duplicate gene-symbol entries were collapsed by retaining the entry with the highest mean expression across samples, and genes not shared across the included datasets were excluded from the combined matrix. Only untreated or baseline A549 samples were included for the A549 group. Genes with missing values or zero variance across samples were removed before principal component analysis (PCA). PCA was performed in R (version 4.2.1) on the curated gene-by-sample matrix. Genes ranking within the top 20% of absolute loading values for PC1 and PC2 were extracted, and their expression patterns were visualized by heatmap after row-wise Z-score scaling. Gene Ontology biological process analysis was performed on the PC1- and PC2-loading gene sets to assist interpretation of the transcriptional structure captured by the PCA. These analyses were used to describe relative transcriptomic positioning and enriched functional themes, not to assign developmental identity or lineage equivalence to A549 cells.

### 2.2. Cell Culture

A549 cells (Procell, CL-0016, Wuhan, China) were cultured in DMEM supplemented with 10% fetal bovine serum, 1× MEM Non-Essential Amino Acids Solution, 1× L-glutamine (Gibco, Carlsbad, CA, USA), and 1× penicillin-streptomycin solution (Solarbio, Beijing, China). Cells were maintained at 37 °C in a humidified incubator with 5% CO_2_ and passaged at approximately 80–90% confluence. The A549 cells used in this study were accompanied by a supplier-provided short tandem repeat (STR) authentication report and were handled as a human lung adenocarcinoma-derived epithelial cell line suitable for reporter-based promoter assays. The supplier-provided STR report was used to verify cell-line identity (Supplementary File S1).

### 2.3. siRNA-Mediated Knockdown

A549 cells were seeded in six-well plates and transfected with small interfering RNAs targeting FOXA2 or SOX9, or with a non-targeting control siRNA, using Lipofectamine™ 3000 Transfection Reagent (Invitrogen, Carlsbad, CA, USA) according to the manufacturer’s instructions. The final siRNA concentration was 50 nM. Cells were harvested 48 h after transfection for immunoblotting analysis. The siRNA sequences used in this study were as follows: human FOXA2 siRNA Sense: 5′-UGAACGGCAUGAACACGUATT-3′, Antisense: 5′-UACGUGUUCAUGCCGUUCATC-3′; human SOX9 siRNA Sense: 5′-CGUGUGAUCAGUGUGCUAATT-3′, Antisense: 5′-UUAGCACACUGAUCACACGTT-3′; negative control Sense: 5′-UUCUCCGAACGUGUCACGUTT-3′, Antisense: 5′-ACGUGACACGUUCGGAGAATT-3′. Knockdown efficiency was assessed by immunoblotting for FOXA2 or SOX9, with GAPDH used as the loading control. The knockdown experiments were used to assess whether FOXA2 or SOX9 perturbation was associated with TP63 isoform-associated protein output. Because one siRNA sequence was used for each target gene, potential siRNA-specific off-target effects were considered when interpreting these data.

### 2.4. Immunoblotting

A549 cells were lysed in RIPA buffer (Sangon, C500005, Shanghai, China) supplemented with phosphatase and protease inhibitors (Beyotime, P1045, Shanghai, China). Protein concentrations were determined using a BCA Protein Assay Kit (Sangon, C503021, Shanghai, China). Equal amounts of protein lysates were denatured in loading buffer, separated by SDS-PAGE, and transferred onto PVDF membranes. After blocking with 5% non-fat milk in TBST, the membranes were incubated with primary antibodies overnight at 4 °C, followed by incubation with HRP-conjugated secondary antibodies for 2 h at room temperature. Protein signals were visualized using an enhanced chemiluminescence substrate and imaged with an iBright^®^ FL 1500 Imaging System (Thermo Scientific, Carlsbad, CA, USA). For same-membrane detection of target proteins and GAPDH, species-distinct primary antibodies were co-incubated where applicable, followed by simultaneous incubation with HRP-conjugated anti-rabbit and anti-mouse secondary antibodies and chemiluminescent detection. GAPDH was used as the loading control, and band intensities were normalized to the corresponding GAPDH signal from the same membrane and lane.

For siRNA knockdown experiments, FOXA2, SOX9, TAp63, and ΔNp63 band intensities were normalized to the corresponding GAPDH signal from the same lane. Relative protein levels were then normalized to the si-Ctrl group, which was set to 1. The ΔNp63/TAp63 ratio was calculated for each matched biological replicate using GAPDH-normalized ΔNp63 and TAp63 values, and the resulting ratio was then normalized to the si-Ctrl group.

The following primary antibodies were used: FOXA2 (1:500, Proteintech, 22474-1-AP, Rosemont, CA, USA), NKX2-1/TTF-1 (1:500, Abcam, ab133638, Cambridge, UK), SOX9 (1:500, Proteintech, 67439-1-Ig, Rosemont, CA, USA), TAp63 (1:500, Novus Biologicals, NBP3-11703, Centennial, CO, USA), and ΔNp63/p40 (1:500, Abcam, ab203826, Cambridge, UK). FOXA2, NKX2-1/TTF-1, and ΔNp63/p40 primary antibodies are rabbit antibodies, whereas SOX9 and TAp63 primary antibodies are mouse antibodies. Mouse anti-GAPDH (1:1000, Beyotime, Shanghai, AF5009, China) was used with rabbit primary antibodies, and rabbit anti-GAPDH (1:1000, Beyotime, AF1186, Shanghai, China) was used with mouse primary antibodies. The following HRP-conjugated secondary antibodies were used: HRP-labeled goat anti-rabbit IgG (H + L) (1:1000, Beyotime, A0208, Shanghai, China) and HRP-labeled goat anti-mouse IgG (H + L) (1:1000, Beyotime, A0216, Shanghai, China).

### 2.5. Immunofluorescence

A549 cells were seeded in confocal culture dishes and cultured to approximately 80% confluence. After washing with PBS, the cells were fixed with 4% paraformaldehyde for 15 min at room temperature, followed by blocking and permeabilization with 5% normal goat serum and 0.3% Triton X-100 in PBS for 1 h. The cells were then incubated with primary antibodies overnight at 4 °C and with fluorophore-conjugated secondary antibodies for 1 h at room temperature in the dark. Nuclei were counterstained with DAPI-containing anti-fade mounting medium (Solarbio, S2110, Beijng, China). Fluorescence images were acquired using a Leica TCS SP8 laser-scanning confocal microscope (Leica Microsystems CMS GmbH, Mannheim, Germany) and analyzed with Leica LAS X 4.7. For quantification, the mean fluorescence intensities of TAp63 and ΔNp63/p40 were normalized to the mean DAPI intensity in the corresponding field.

The following primary antibodies were used: anti-TAp63 (1:500, Novus Biologicals, NBP3-11703, Centennial, CO, USA; mouse monoclonal) and anti-ΔNp63/p40 (1:500, Abcam, ab203826, Cambridge, UK; rabbit monoclonal). The following secondary antibodies were used: goat anti-mouse IgG H&L Alexa Fluor 594 (1:500, Abcam, ab150116, Cambridge, UK; rabbit monoclonal) and goat anti-rabbit IgG H&L Alexa Fluor 488 (1:500, Abcam, ab150077, Cambridge, UK; rabbit monoclonal). DAPI is shown in blue, TAp63 in red, and ΔNp63/p40 in green.

### 2.6. Isolation of Rabbit Genomic DNA

Genomic DNA was extracted from rabbit embryonic fibroblasts (REFs) (kindly provided by Xiaojing Zhang, Second Affiliated Hospital of Fujian Medical University). REFs were seeded in a 100 mm dish and cultured until 80% confluence was reached. The media was aspirated, and the cells were washed twice with PBS. A mixture of 1250 µL 2× lysis buffer (10% SDS, 0.5 M EDTA, 1 M Tris, pH 7.5), 1000 µL PBS, and 25 µL proteinase K (20 mg/mL) was added to the dish, which was then sealed with parafilm and incubated overnight at 50 °C. The lysate was subjected to phenol/chloroform extraction, and genomic DNA was precipitated using 7.5 M NH4Acetate and isopropanol. The DNA pellets were dissolved in TE buffer for subsequent experiments.

### 2.7. Cloning Both Distal and Proximal Promoters of Rabbit TP63 Gene

Based on BLAST (version 2.17.0+; NCBI, Bethesda, MD, USA) comparison of the rabbit TP63 locus (Oryctolagus cuniculus, NC_013682.1) with the corresponding human (Homo sapiens, NC_000003.12) and mouse (Mus musculus, NC_000082.7) loci, locally homologous promoter-associated segments were identified within the TAp63- and ΔNp63-associated regions. Rabbit genomic fragments encompassing these homologous segments together with adjacent upstream or flanking sequences were amplified using Platinum™ SuperFi™ II PCR Master Mix (Invitrogen, Carlsbad, CA, USA) and cloned into the pCR2.1 vector (Invitrogen, Carlsbad, CA, USA) (Table 1). Clones were selected and sequenced to confirm insert fidelity before subcloning into the reporter vector.

### 2.8. Constructing Promoter Reporter Vectors

After sequencing to verify clone fidelity, full-length distal and proximal promoters were constructed into the pGL4.17 vector (Promega, Madison, WI, USA) between the *KpnI* and *NheI* sites using T4 ligase (Promega, Madison, WI, USA) to generate correctly oriented D and P reporter vectors.

For promoter deletion analysis, six genomic DNA fragments were generated by sequentially truncating the 5′ end of both full-length promoters using PCR amplification with specific primer sets (Invitrogen, Carlsbad, CA, USA) (Table 1) and validated by gel electrophoresis (Supplementary Figure S1). These fragments were then cloned into pGL4.17 using T4 ligase (Promega, Madison, WI, USA).

For site-directed mutagenesis, putative transcription factor binding sites (TFBSs) were predicted using the online software JASPAR 2026 and compared with published TF binding sequences (Table 2 and Table S1). The predicted TFs included FOXA1/2, NKX2-1, SOX2, SOX9, and TP63, which were selected because of their reported involvement in epithelial lineage programs, lung developmental regulatory networks, or TP63-associated epithelial biology. Site-directed mutations were introduced into both truncation constructs and full-length promoters using a mutagenesis kit (Vazyme, Shanghai, China).

After construction, all reporter vectors were verified for insert orientation and site-directed mutations by sequencing.

### 2.9. Transient Transfection of Luciferase Assay

A549 cells were seeded in 96-well cell culture plates and grown for 24 h to 90% confluence for transient transfection of reporter plasmids. Transfection was performed using Lipofectamine™ 3000 Transfection Reagent (Invitrogen, Carlsbad, CA, USA) according to the manufacturer’s instructions. Briefly, the culture media was replaced with Opti-MEM (Gibco, Carlsbad, CA, USA) before transfection. Each well was co-transfected with 80 ng of reporter plasmid and 10 ng of pGL4.75 (Promega, Madison, WI, USA). Transfections were performed in quadruplicate across at least three independent experiments. Firefly and Renilla luciferase activities were measured using the Dual-Glo Luciferase Assay system (Promega, E2920, Madison, WI, USA) following the manufacturer’s protocol. Specifically, 16 h post-transfection, 75 µL of Dual-Glo Luciferase Assay Reagent was added to each well, and the plate was incubated at 25 °C for 10 min with gentle shaking. Firefly luminescence was measured at 10 s per well using a Microplate Fluorometer and Luminometer (Thermo Scientific™ Fluoroskan™, Waltham, MA, USA). Subsequently, 75 µL of Dual-Glo Stop & Glo Reagent was added, and after the same incubation conditions, Renilla luminescence was measured at 10 s per well. After subtracting the background values, the Firefly/Renilla luminescence ratio for each well was calculated. Promoter activity was determined by the Firefly/Renilla luciferase intensity ratio for each construct, normalized to the luciferase activity of the pGL4.17 vector, and expressed as fold activation.

### 2.10. Chromatin Immunoprecipitation-qPCR (ChIP-qPCR)

Chromatin immunoprecipitation (ChIP) assays were performed using the Pierce™ Magnetic ChIP Kit (Thermo Scientific, # 26157, Waltham, MA, USA) following the manufacturer’s instructions with minor modifications. Briefly, A549 cells were seeded onto 10 cm dishes and cultured to ~90% confluency. Cells were then crosslinked with formaldehyde, lysed, and chromatin was sheared by sonication to fragments of approximately 100–200 bp. For each ChIP reaction, 10 μg of chromatin was incubated overnight with 4 μg of transcription factor-specific antibodies. Normal rabbit IgG (Yeasen, 36113ES10, Shanghai, China) and normal mouse IgG (Yeasen, 36111ES60, Shanghai, China) were used as host-matched negative controls for rabbit- and mouse-derived ChIP antibodies, respectively. Immunoprecipitated DNA was purified and analyzed using a quantitative PCR kit (Agbio, AG11734, Changsha, China) with primers targeting the indicated human TP63 promoter-associated regions, which were designed based on the human TP63 genomic sequence and verified for specificity using NCBI BLAST (Table 3). Quantitative analysis was performed using both the % Input method and fold enrichment over the corresponding host-matched IgG control. In addition, qPCR amplicons were resolved by 2% agarose gel electrophoresis and visualized using ethidium bromide staining. These ChIP-qPCR assays were designed to test enrichment at selected candidate TP63 promoter-associated regions. ChIP-qPCR enrichment was calculated relative to the corresponding host-matched IgG controls and is reported as relative enrichment at the selected TP63 promoter-associated regions.

The following primary antibodies were used in this study: FOXA2 (Abcam, ab108396, Cambridge, UK), NKX2-1 (Thermo Scientific, MA5-13961, Waltham, MA, USA), SOX9 (MCE, HY-P80335, Monmouth Junction, NJ, USA), and TP63 (CST, #13109, Danvers, MA, USA).

### 2.11. Statistical Analysis

All quantitative data are presented as means ± SD from independent biological replicates unless otherwise indicated. Statistical analyses were applied to dual-luciferase promoter reporter assays, ChIP-qPCR quantification, and immunoblot densitometry. For promoter reporter assays, relative promoter activity or fold activation was analyzed using Welch’s one-way analysis of variance followed by Games–Howell multiple comparison tests. For ChIP-qPCR, % Input values were calculated for each independent ChIP experiment, and fold enrichment over the corresponding host-matched IgG control was used to present relative transcription-factor-associated enrichment at the indicated TP63 promoter regions. When multiple promoter regions or antibody groups were compared, Welch’s one-way ANOVA followed by Games–Howell tests was used. Agarose gel images of qPCR amplicons were used as qualitative confirmation and were not included in statistical testing. For siRNA knockdown experiments, immunoblot band intensities were quantified from independent biological replicates after normalization to GAPDH. Two-group comparisons used for knockdown validation were analyzed using Welch’s *t*-test. Comparisons among si-Ctrl, si-FOXA2, and si-SOX9 groups were analyzed using Welch’s one-way ANOVA followed by Games–Howell multiple comparison tests. Statistical analyses were performed using SPSS Statistics version 26, and *p* < 0.05 was considered statistically significant.

## 3. Results

### 3.1. Deletion Mapping and Motif-Level Perturbation Identify Candidate Regulatory Elements Within TP63 Distal and Proximal Promoter Reporters

To define promoter-associated reporter fragments for rabbit TP63, we compared the rabbit TP63 locus (Oryctolagus cuniculus, NC_013682.1) with corresponding human (Homo sapiens, NC_000003.12) and mouse (Mus musculus, NC_000082.7) TP63 loci using NCBI BLAST.

For the TAp63-associated distal region, locally alignable segments were identified in the corresponding human and mouse promoter neighborhoods, with local sequence identities of approximately 79% and 82%, respectively. These alignable segments overlapped the region corresponding to exon 1 of the rabbit TAp63 transcript. We therefore cloned a 2226 bp rabbit genomic fragment encompassing the alignable segment and adjacent upstream sequence as an operational distal promoter-associated reporter region. Sanger sequencing confirmed a 2227 bp insert spanning −2138 to +89 relative to the translation start site, which was defined as the full-length distal reporter construct (D) for deletion analysis.

For the ΔNp63-associated proximal region, the rabbit fragment was selected primarily on the basis of its position relative to the annotated ΔNp63-associated transcript-initiation/proximal promoter neighborhood and the presence of locally alignable segments in the corresponding human and mouse TP63 loci. These local alignments were not uniform across the entire region. Instead, discrete subsegments showed variable similarity, with some human-rabbit or mouse-rabbit local alignments falling within the approximate 69–95% identity range, depending on the specific site examined. In addition, the corresponding interval in the rabbit reference genome contained unresolved “N” gaps. We therefore cloned a broader 3442 bp rabbit genomic fragment spanning the candidate proximal promoter neighborhood and flanking sequence instead of relying on a single short conserved block. Sequencing confirmed a 3459 bp insert spanning −3353 to +100 relative to the translation start site, which was operationally defined as the full-length proximal reporter construct (P). Thus, the proximal rabbit reporter was defined as a positionally selected promoter-neighborhood construct containing locally alignable subsegments across the examined mammalian TP63 loci.

**Figure 1:**
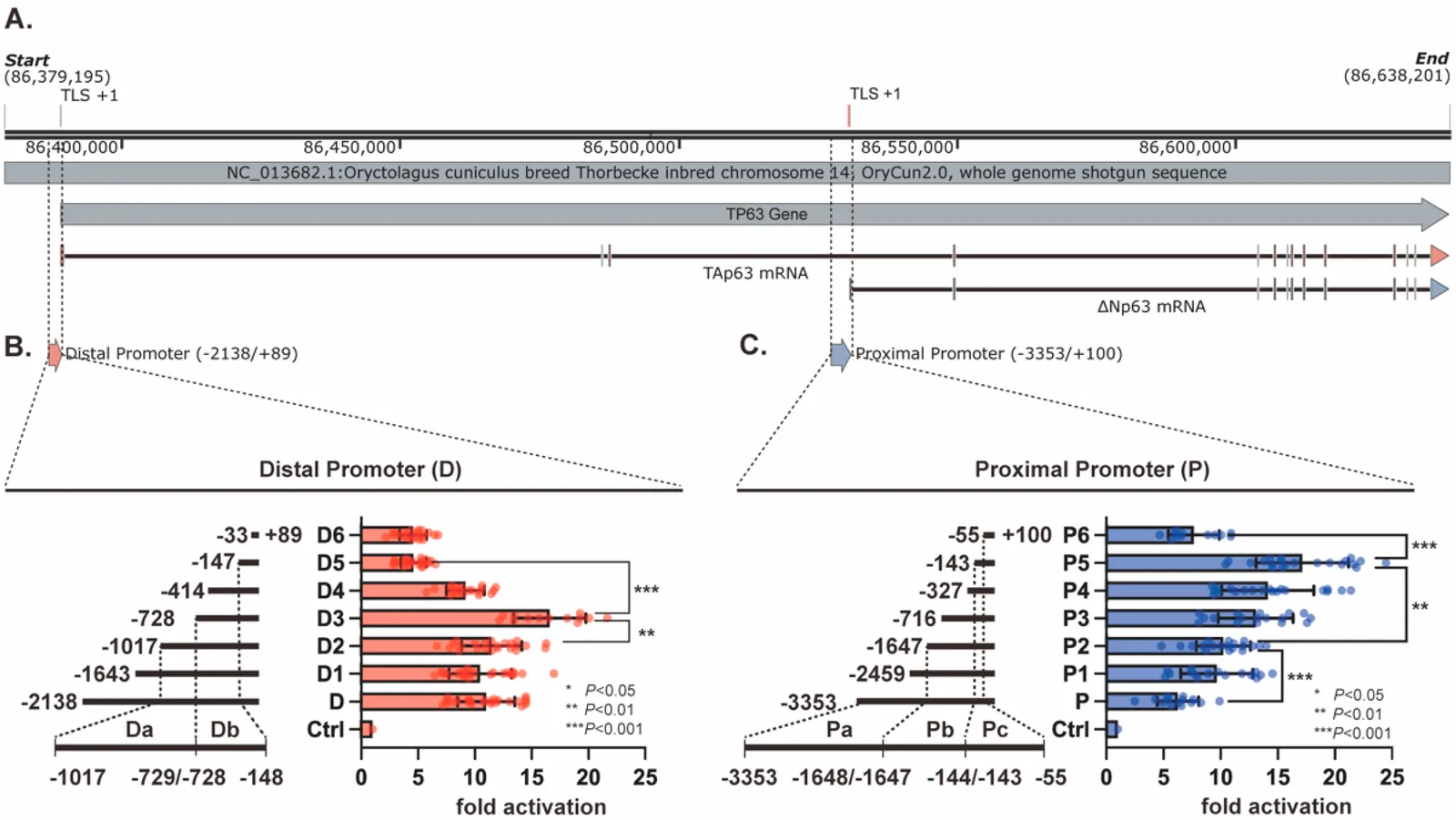
Deletion mapping of rabbit TP63 distal and proximal promoter reporters in A549 cells. (**A**) Schematic overview of the rabbit TP63 locus, showing the rabbit TP63 distal promoter associated with the TAp63 transcript and the proximal promoter associated with the ΔNp63 transcript. (**B**) Serial 5′ truncations of the distal promoter reporter, all sharing a common 3′ end at +89 relative to the translation start site, and their luciferase activities in A549 cells. The deletion profile identifies the −1107 to −729 interval as a candidate negative regulatory segment and the −728 to −148 interval as a candidate positive regulatory segment in this reporter context. (**C**) Serial 5′ truncations of the proximal promoter reporter, all sharing a common 3′ end at +100, and their luciferase activities in A549 cells. The deletion profile identifies upstream segments (−3353 to −144) as candidate negative regulatory regions and the −143 to −55 interval as a candidate positive regulatory segment in this reporter context. Luciferase activities were normalized to Renilla and are shown as fold change relative to the empty pGL4.17 vector (Ctrl = 1). Statistical comparisons were performed as described in the Methods section, with the corresponding multiple-comparison procedures applied according to the experimental design. Data are presented as mean ± SEM from at least three independent biological replicates. * *p* < 0.05, ** *p* < 0.01, *** *p* < 0.001. Throughout the figures, red indicates distal promoter-associated constructs or regions, whereas blue indicates proximal promoter-associated constructs or regions.

To delineate the reporter regions that contribute most strongly to rabbit TP63 promoter activity, we cloned the full-length distal (−2138/+89) and proximal (−3353/+100) fragments, together with a series of 5′ deletions, into the pGL4.17 luciferase vector (Figure 1A–C and Table 1). The successful generation of these reporter plasmids was verified by double-enzyme digestion and agarose gel electrophoresis (Supplementary Figure S1). In the A549-based reporter assay, both full-length fragments were transcriptionally active, supporting their use for promoter-level dissection in this transfection-compatible human epithelial background.

Within the rabbit TP63 distal promoter series, luciferase activity was similar in D1 (−1689/+89) and D2 (−1107/+89), reached its maximum in D3 (−728/+89), and then dropped markedly in D4 (−414/+89) and D5 (−147/+89), with little additional change in D6 (−3/+89) (Figure 1B). This pattern indicates that the −728 to −148 interval contributes positively to reporter output, whereas the upstream −1107 to −729 interval exerts a restraining effect in the truncation series. Within the rabbit TP63 proximal promoter series, activity increased from the full-length construct to P2 (−2770/+100) and rose further through P3, P4, and P5 (−143/+100), before declining in P6 (−55/+100) (Figure 1C). These data localize a positive regulatory contribution to the −143 to −55 interval, while the more upstream segments appear to reduce reporter activity in this assay format. On the basis of these deletion profiles, we defined Da/Db in the distal promoter and Pa/Pb/Pc in the proximal promoter as candidate regulatory segments for subsequent motif-level analysis.

**Figure 2:**
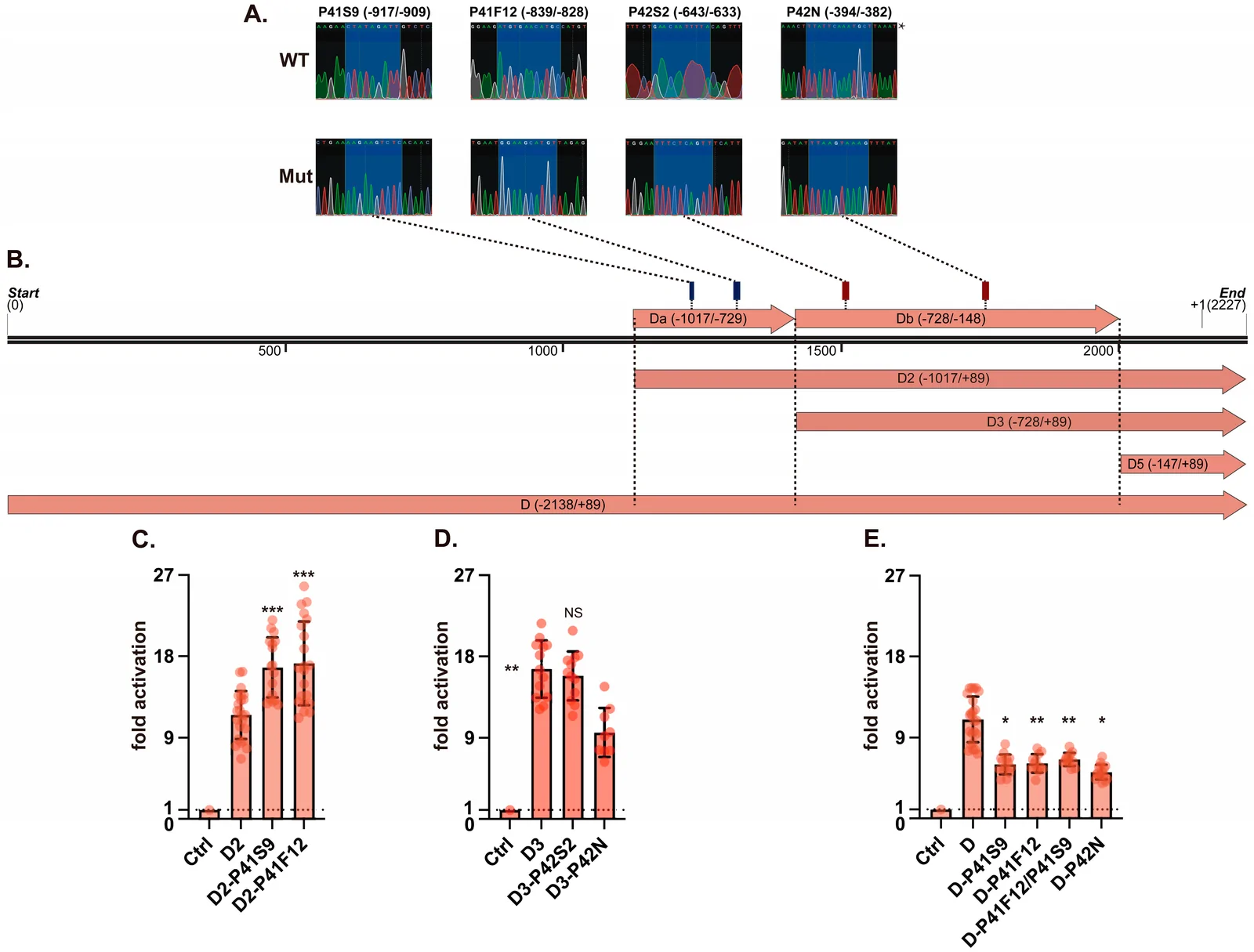
Motif-level perturbation analysis of candidate regulatory elements in the rabbit TP63 distal promoter reporter. (**A**) Representative Sanger sequencing traces confirming site-directed mutagenesis of the indicated distal promoter motifs. The site labels correspond to P41S9 (SOX9), P41F12 (FOXA1/FOXA2), P42S2 (SOX2), and P42N (NKX2-1). An asterisk indicates chromatograms generated using a reverse sequencing primer; the displayed sequence therefore corresponds to the reverse complement of the reference strand. (**B**) Schematic map of the distal promoter reporter constructs showing the positions of the computationally predicted candidate motifs within Da and Db. Dashed guide lines connect the labeled sequencing traces in panel A with their corresponding genomic positions. These motifs were selected as candidate regulatory elements for functional perturbation and are not presented as experimentally validated binding sites. (**C**,**D**) Luciferase activities of the D2 and D3 truncation reporters carrying motif mutations in Da (**C**) and Db (**D**), respectively, compared with the corresponding wild-type constructs. (**E**) Luciferase activities of selected motif mutations tested in the full-length distal promoter reporter. These assays identify candidate motif-dependent regulatory effects within an exogenous rabbit TP63 distal promoter reporter tested in A549 cells. Luciferase activities were normalized to Renilla and are shown as fold change relative to the empty pGL4.17 vector (Ctrl = 1). Statistical comparisons were performed as described in the Methods section, with the corresponding multiple-comparison procedures applied according to the experimental design. Data are presented as mean ± SEM from at least three independent biological replicates. * *p* < 0.05, ** *p* < 0.01, *** *p* < 0.001.

**Figure 3:**
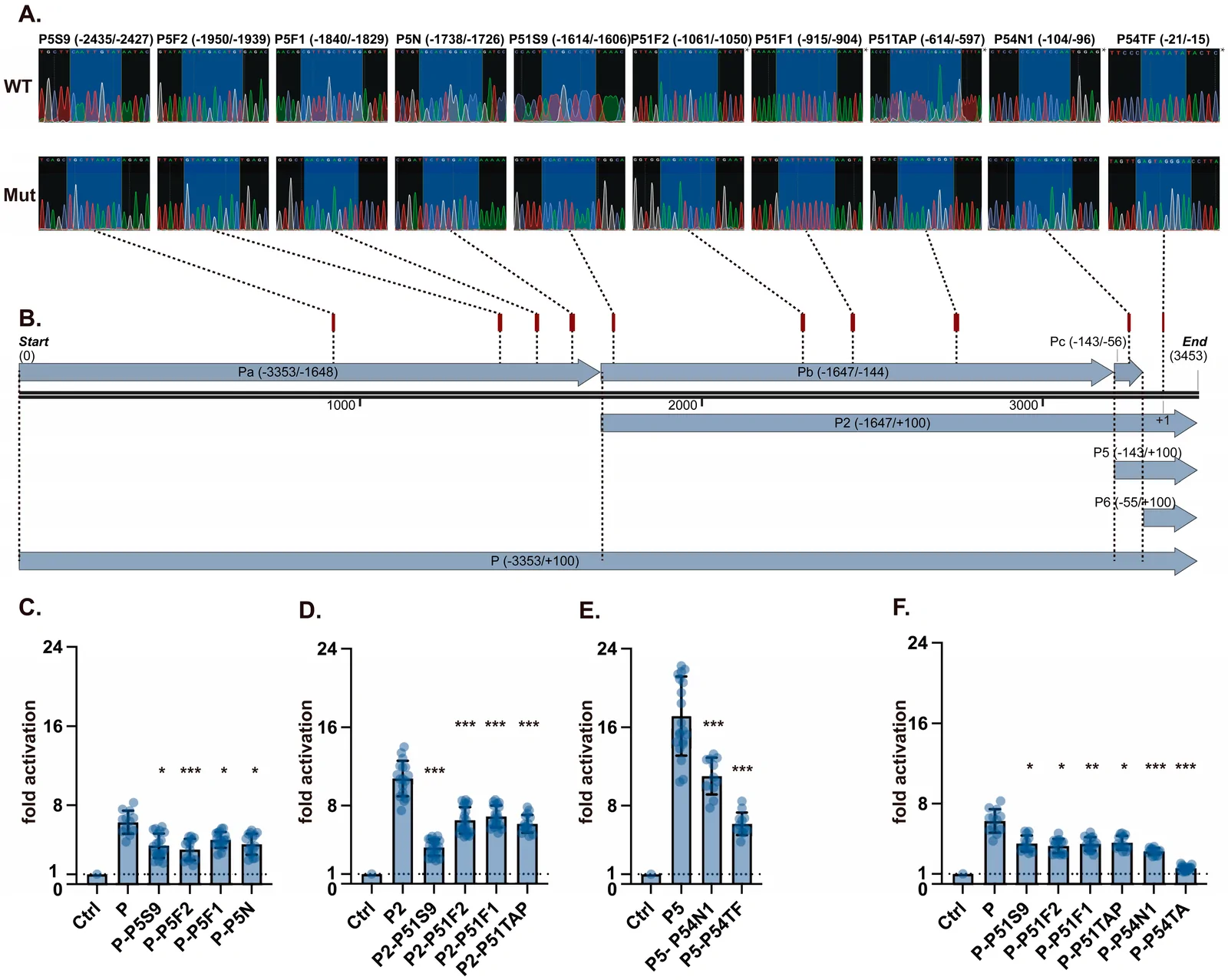
Motif-level perturbation analysis of candidate regulatory elements in the rabbit TP63 proximal promoter reporter. (**A**) Representative Sanger sequencing traces confirming site-directed mutagenesis of the indicated proximal promoter motifs. The site labels correspond to P5S9 (SOX9), P5F2 (FOXA2), P5F1 (FOXA1), P5N (NKX2-1), P51S9 (SOX9), P51F2 (FOXA2), P51F1 (FOXA1), P51TAP (TP63), P54N1 (NKX2-1), and P54TF (TATA-box-associated element). An asterisk indicates chromatograms generated using a reverse sequencing primer; the displayed sequence therefore corresponds to the reverse complement of the reference strand. (**B**) Schematic map of the proximal promoter reporter constructs showing the positions of the computationally predicted candidate motifs within Pa, Pb, and Pc. Dashed guide lines connect the labeled sequencing traces in panel A with their corresponding genomic positions. These motifs were selected as candidate regulatory elements for functional perturbation and are not presented as experimentally validated binding sites. (**C**) Luciferase activities of the full-length proximal reporter carrying selected Pa-region motif mutations. (**D**) Luciferase activities of the P2 truncation reporter carrying selected Pb-region motif mutations. (**E**) Luciferase activities of the P5 truncation reporter carrying selected Pc-region motif mutations. (**F**) Luciferase activities of selected Pb- and Pc-region motif mutations tested in the full-length proximal promoter reporter. These assays identify candidate motif-dependent regulatory effects within an exogenous rabbit TP63 proximal promoter reporter tested in A549 cells. Luciferase activities were normalized to Renilla and are shown as fold change relative to the empty pGL4.17 vector (Ctrl = 1). Statistical comparisons were performed as described in the Methods section, with the corresponding multiple-comparison procedures applied according to the experimental design. Data are presented as mean ± SEM from at least three independent biological replicates. * *p* < 0.05, ** *p* < 0.01, *** *p* < 0.001.

We next asked whether predicted transcription-factor binding sites within these candidate segments contribute to reporter activity. Using JASPAR and published binding motifs, we mapped computationally predicted FOXA1, FOXA2, SOX9, SOX2, NKX2−1, and TP63 candidate binding motifs across the distal Da/Db and proximal Pa/Pb/Pc regions (Figure 2B and Figure 3B, Table 2 and Table S1). Site-directed mutations were introduced into the selected motifs and verified by Sanger sequencing (Figure 2A, Figure 3A and Figure S11A,B) before dual-luciferase testing.

In the rabbit TP63 distal promoter, mutation of the SOX9 and FOXA1/2 motifs in the D2 fragment (−1107/+89) increased reporter activity by approximately 1.5-fold compared with the corresponding wild-type construct, whereas mutation of the SOX2 motif had no significant effect (Figure 2C). In the D3 fragment (−728/+89), disruption of the NKX2-1 site reduced activity by more than 1.5-fold (Figure 2D). When the same sites were tested in the full-length distal promoter, deletion of the NKX2-1 motif again reduced reporter activity, by nearly twofold, whereas mutation of the SOX9 or FOXA1/2 motifs now lowered activity instead of increasing it (Figure 2E). Thus, within the distal rabbit TP63 reporter, NKX2-1 behaved as a reproducible positive input, whereas SOX9 and FOXA1/2 showed promoter-context-dependent effects across truncated and full-length constructs.

In the rabbit TP63 proximal promoter, mutation of the predicted FOXA1/2, SOX9, NKX2-1, TAp63, and TATA-box elements all reduced reporter activity across the tested constructs (Figure 3C–F). The drop associated with TATA-box mutation was especially strong and approached near-complete loss of activity, indicating that this sequence is required for efficient transcription initiation in the proximal reporter context. The effects of the remaining motif mutations were smaller in magnitude but directionally consistent, supporting the view that the proximal rabbit TP63 reporter integrates multiple positive cis-inputs.

Collectively, the deletion and mutagenesis data identify motif-dependent regulatory effects within TP63 distal and proximal promoter reporters and establish a functional framework for promoter-level dissection of TP63 dual-promoter responsiveness.

### 3.2. Endogenous ChIP-qPCR Supports Local Enrichment at Human TP63 Promoter-Associated Regions

To provide context for the promoter regions analyzed in this study, we first examined publicly available chromatin-interaction and regulatory annotation tracks across the human TP63 locus in A549 cells. A broad overview of the TP63 region is shown in Supplementary Figure S2, and enlarged views centered on the TAp63 and ΔNp63 promoter neighborhoods are shown in Supplementary Figure S3 and Supplementary Figure S4, respectively. Public A549 Hi-C maps were visualized in the 3D Genome Browser; ENCODE cCRE annotations with promoter-like signatures (PLS), proximal enhancer-like signatures (pELS), distal enhancer-like signatures (dELS), CTCF-associated elements, and RefSeq gene models were displayed as accompanying regulatory and gene-annotation tracks [24,25,26]. These public datasets provide local chromatin-context information for interpreting the genomic neighborhoods of the selected TP63 promoter-associated regions in A549 cells. They were used to provide additional genomic context alongside the endogenous ChIP-qPCR analyses performed in this study.

We next aligned the TP63 promoter regions analyzed in this study across human, mouse, and rabbit. The distal regions corresponding to P41F12, P42N, and P41S9 (Supplementary Figure S5A–C), and the proximal regions corresponding to P5F2, P51F2, P5N, P54N1, P5S9, P51S9, and P51TAP (Supplementary Figure S6A–G), could be located within the corresponding TP63 promoter intervals in the three species. Sequence similarity was variable across sites, with multiple mismatches and short gaps in several regions. These alignments provide sequence-context information for the promoter regions selected for reporter and ChIP-qPCR analysis.

**Figure 4:**
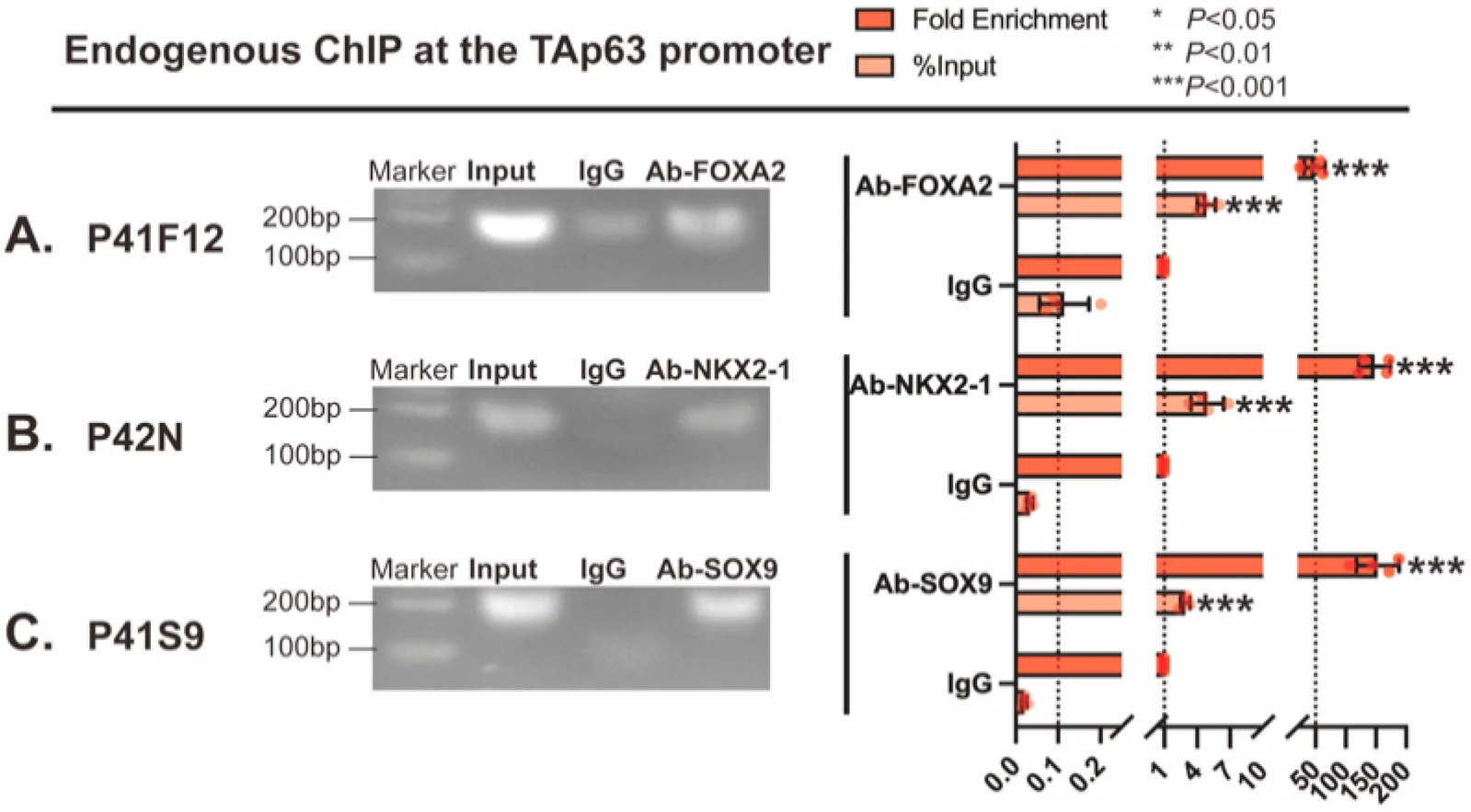
Endogenous ChIP-qPCR analysis of transcription-factor-associated enrichment at human TAp63 promoter regions in A549 cells. (**A**) FOXA2 enrichment at P41F12, (**B**) NKX2-1 enrichment at P42N, (**C**) SOX9 enrichment at P41S9 at the corresponding human TAp63 promoter-associated region. For each site, quantitative data are shown as % input and fold enrichment relative to the corresponding host-matched IgG controls, together with representative agarose gel images of the corresponding amplicons. * *p* < 0.05, ** *p* < 0.01, *** *p* < 0.001.

**Figure 5:**
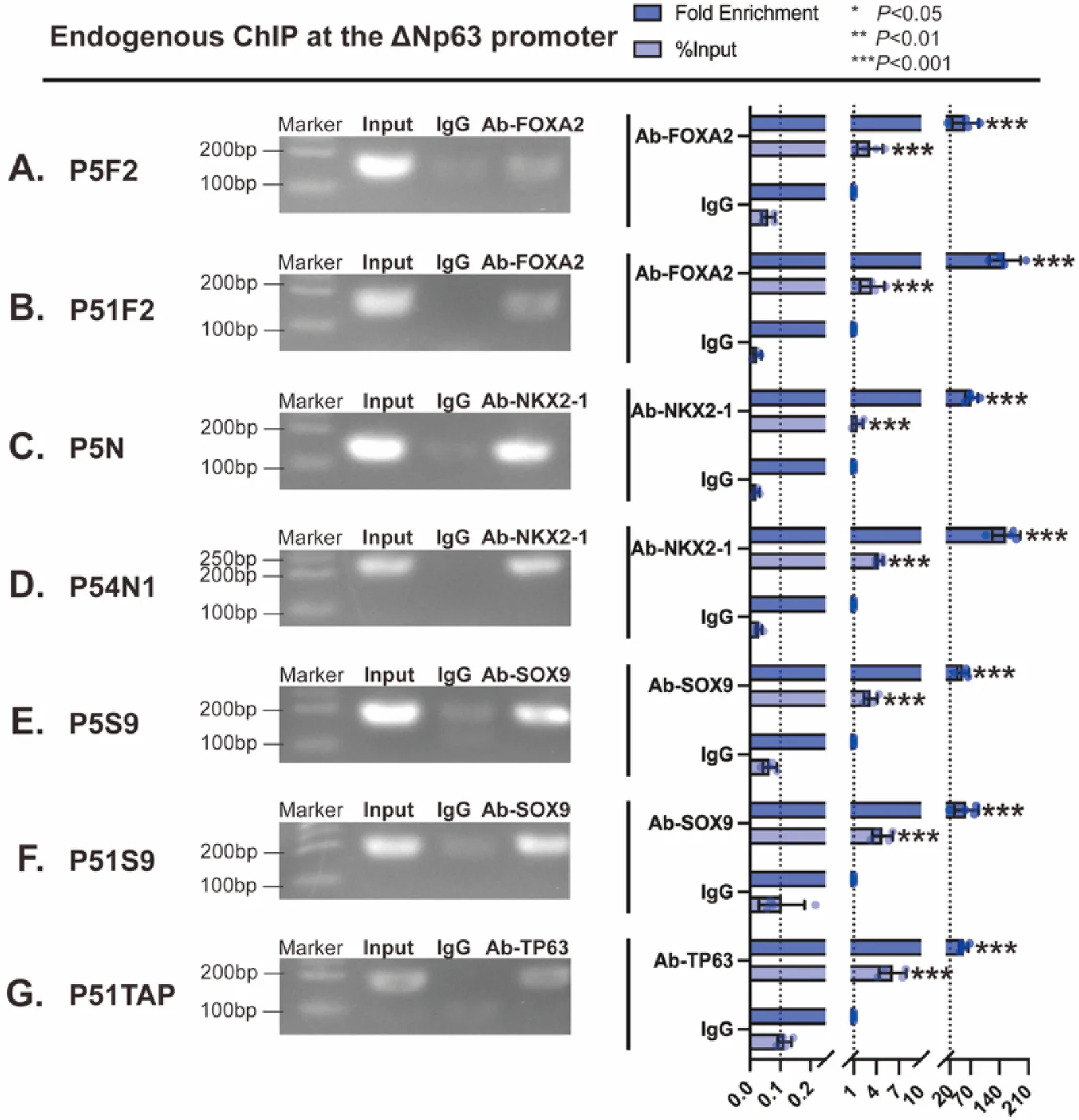
Endogenous ChIP-qPCR analysis of transcription-factor-associated enrichment at human ΔNp63 promoter regions in A549 cells. (**A**,**B**) FOXA2 enrichment at P5F2 and P51F2, (**C**,**D**) NKX2-1 enrichment at P5N and P54N1, (**E**,**F**) SOX9 enrichment at P5S9 and P51S9, (**G**) TP63 enrichment at P51TAP at the corresponding human ΔNp63 promoter-associated region. Quantitative data are shown as % input and fold enrichment relative to the corresponding host-matched IgG controls, together with representative agarose gel images of the corresponding amplicons. * *p* < 0.05, ** *p* < 0.01, *** *p* < 0.001.

After establishing this locus and sequence context, we performed endogenous ChIP-qPCR in A549 cells to examine transcription-factor-associated enrichment at the corresponding human TAp63 and ΔNp63 promoter regions. The primer sets used for ChIP-qPCR are listed in Table 3. Within the TAp63 promoter region, endogenous ChIP-qPCR detected enrichment relative to the corresponding host-matched IgG controls of the tested transcription factors at the analyzed sites (Figure 4A–C). FOXA2 was most strongly enriched at P41F12, with approximately 50-fold enrichment over IgG and % input values of about 4.8% (Figure 4A). NKX2-1 and SOX9 were enriched at P42N and P41S9, respectively, with fold enrichment values ranging from approximately 107-fold to 188-fold and % input values reaching about 6.9% (Figure 4B,C). The ΔNp63 promoter region showed broader but still site-dependent enrichment across the analyzed sites (Figure 5A–G). FOXA2 was enriched at P5F2 and P51F2, with fold enrichment values of approximately 15-fold to 204-fold (Figure 5A,B). NKX2-1 enrichment was detected at P5N and P54N1, ranging from about 55-fold to 180-fold (Figure 5C,D). SOX9 enrichment at P5S9 and P51S9 ranged from about 19-fold to 83-fold (Figure 5E,F), and TP63 itself was enriched by approximately 68-fold at P51TAP (Figure 5G). The corresponding gel bands are shown in Figure 4 and Figure 5, and the full gel images are provided in Supplementary Figure S7.

Taken together, the public chromatin-context panels, sequence alignments, and endogenous ChIP-qPCR assays provide local evidence that selected FOXA2-, NKX2-1-, SOX9-, and TP63-associated signals are enriched at TP63 promoter-associated regions in the A549 cells used here. These findings connect the reporter-defined candidate sites with corresponding human genomic regions and support the promoter-centered interpretation of the functional assays.

### 3.3. FOXA2 or SOX9 Knockdown Shifts Endogenous TP63 Isoform-Associated Protein Balance in A549 Cells

**Figure 6:**
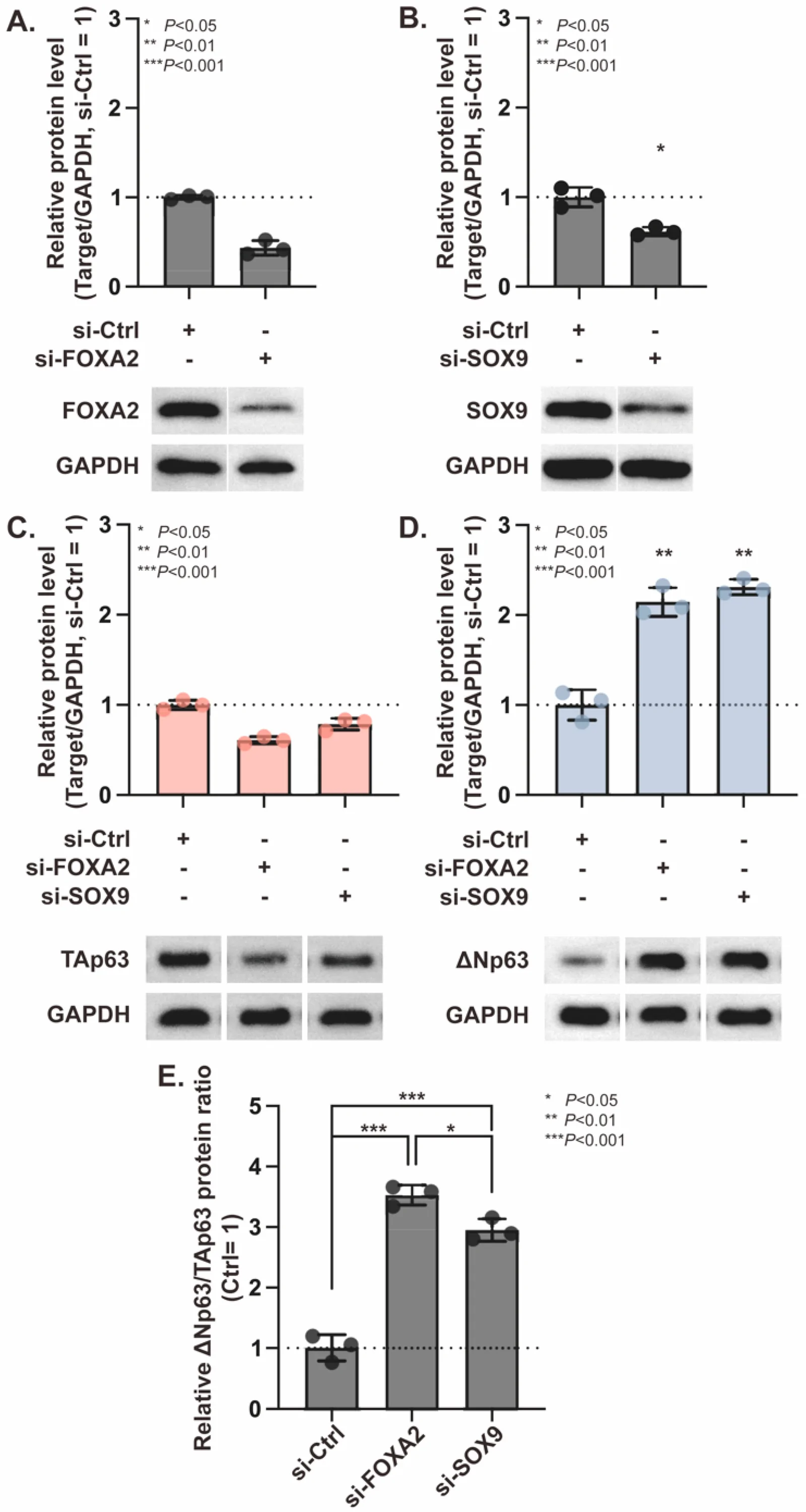
FOXA2 or SOX9 knockdown shifts the endogenous TAp63/ΔNp63 protein balance in A549 cells. (**A**,**B**) FOXA2 and SOX9 knockdown in A549 cells. Band intensities were normalized to GAPDH and then to the si-Ctrl group. (**C**,**D**) Effects of FOXA2 or SOX9 knockdown on endogenous TAp63 and ΔNp63 protein levels. (**E**) Relative ΔNp63/TAp63 protein ratio after FOXA2 or SOX9 knockdown. These findings provide supportive endogenous evidence consistent with the promoter-context-dependent regulatory model proposed in this study, in which transcription-factor-associated inputs influence TP63 isoform-associated output. The study-wide conceptual model integrating these observations with the promoter-level findings is summarized in the Graphical Abstract. Data are presented as mean ± SD from three independent biological replicates. Two-group comparisons used for knockdown validation were analyzed using Welch’s *t*-test. Comparisons among si-Ctrl, si-FOXA2, and si-SOX9 groups were analyzed using Welch’s one-way ANOVA followed by Games–Howell tests. * *p* < 0.05, ** *p* < 0.01, *** *p* < 0.001.

We next examined whether perturbation of FOXA2 or SOX9 was associated with endogenous TP63 isoform-related protein output in A549 cells. These two factors were selected because they were detectable in the present assay system and showed motif-associated reporter effects. A549 cells were therefore transfected with siRNAs targeting FOXA2 or SOX9, followed by immunoblotting analysis. FOXA2 and SOX9 protein levels were efficiently reduced by their corresponding siRNAs compared with the si-Ctrl group, confirming effective knockdown under the experimental conditions (Figure 6A,B). Full-membrane immunoblot images corresponding to these cropped panels are provided in Supplementary Figure S8.

Knockdown of either FOXA2 or SOX9 reduced endogenous TAp63 protein abundance (Figure 6C). In contrast, ΔNp63 protein levels increased after FOXA2 or SOX9 knockdown (Figure 6D). As a result, the relative ΔNp63/TAp63 protein ratio was markedly increased in both knockdown groups, with a stronger shift observed after FOXA2 depletion than after SOX9 depletion (Figure 6E).

These data indicate that FOXA2 or SOX9 perturbation is associated with altered endogenous TP63 isoform-associated protein abundance in A549 cells. The reduction in TAp63 after FOXA2 or SOX9 knockdown is directionally consistent with the positive contribution of FOXA/SOX9-responsive motifs observed in the full-length distal promoter reporter context. The increase in ΔNp63 after FOXA2 or SOX9 depletion suggests that intact-cell TP63 isoform-associated protein balance may involve indirect transcriptional network effects, compensatory regulation, post-transcriptional regulation, or differential protein stability.

### 3.4. Transcriptomic and Protein-Level Contextualization of the A549 Assay Background

**Figure 7:**
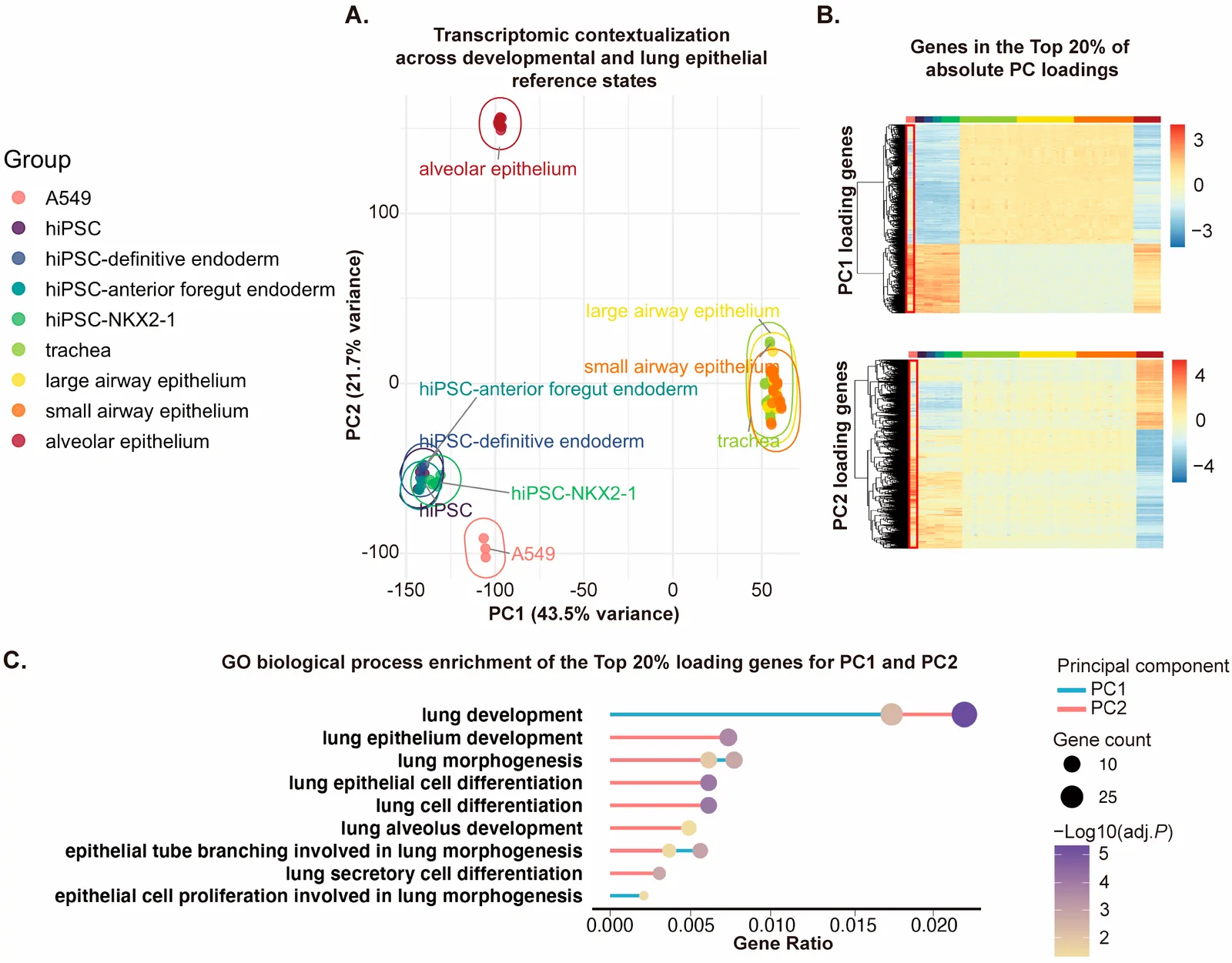
Transcriptomic positioning of A549 cells relative to developmental and respiratory epithelial references. (**A**) Principal component analysis (PCA) of GEO-derived gene expression datasets. PC1 (43.5% variance) separates hiPSC-derived endodermal/intermediate references from mature airway epithelial samples, whereas PC2 (21.7% variance) distinguishes the alveolar compartment. A549 forms a discrete cluster that remains separated from both developmental intermediates and primary respiratory epithelial reference populations. (**B**) Heatmaps of genes within the top 20% of absolute loadings for PC1 and PC2. A549 (red box) shows coordinated loading-gene expression features that partially overlap with developmental and respiratory epithelial references, while retaining a distinct overall transcriptional pattern. (**C**) Gene Ontology biological process analysis of the top 20% absolute loading genes for PC1 and PC2. Lung-related GO terms extracted from the enrichment results are shown to provide biological context for the PCA structure. Because no formal cross-dataset batch correction was performed, the PCA was interpreted descriptively and was not used to infer lineage equivalence.

To contextualize the A549-based promoter assays, we analyzed GEO-derived expression datasets representing hiPSC-derived developmental stages, primary respiratory epithelial populations, and untreated A549 cells (Supplementary Table S2). PCA showed that A549 formed a distinct cluster separated from developmental and primary epithelial reference groups (Figure 7A), while loading-gene heatmaps and lung-related GO terms showed partial overlap with epithelial and respiratory-associated features (Figure 7B,C and Supplementary Tables S3 and S4). These data support the use of A549 as a uniform, transfection-compatible epithelial cancer-derived assay background for promoter-reporter interrogation, while indicating that it should not be treated as a transcriptional equivalent of primary or developmental lung epithelial populations. Immunoblotting detected FOXA2, SOX9, TP63 isoform-associated proteins, and NKX2-1/TTF-1 signal in the STR-authenticated A549 cells used here, and immunofluorescence detected TAp63 and ΔNp63 signals (Supplementary Figures S9 and S10).

## 4. Discussion

This study functionally dissects TP63 distal TAp63-associated and proximal ΔNp63-associated promoter regions using a reporter-based cell-culture system. Serial deletion and motif-level perturbation identified promoter intervals and FOXA1/2-, NKX2-1-, SOX9-, and TP63-related motifs that modulate reporter output in a promoter-context-dependent manner (Figure 1, Figure 2, Figure 3 and Figure S1; Table 1, Table 2 and Table S1). Endogenous ChIP-qPCR at corresponding human TP63 promoter-associated regions provided local enrichment evidence for selected transcription-factor-associated signals (Figure 4, Figure 5 and Figures S2–S7; Table 3), while FOXA2 or SOX9 perturbation shifted endogenous TAp63 and ΔNp63 protein abundance (Figure 6 and Figure S8). These data support a promoter-level model in which TP63 dual-promoter output is influenced by promoter architecture, motif composition, and cellular regulatory background within the experimental system used here.

This interpretation is consistent with established TP63 biology. TP63 encodes multiple isoforms, including TAp63 and ΔNp63, through alternative promoter usage and N-terminal configuration [1]. In the respiratory epithelium, TP63 is linked to embryonic progenitor and postnatal basal-cell programs, and TP63-positive cells contribute to regionally distinct airway stem and repair-associated cell populations [2,3,4,5,6,7]. These studies provide the biological rationale for examining TP63 promoter regulation in epithelial contexts. The present study addresses this question at the promoter-reporter level and provides a functional entry point for future work in primary, organoid, or developmental models.

The A549 cellular context is central to the interpretation of these data. A549 cells are useful for promoter-reporter experiments because they are human, epithelial, readily transfectable, experimentally uniform, and widely used in respiratory and lung cancer research [27]. In the present study, STR authentication supports the identity of the A549 cells used for the assays. Nevertheless, STR authentication verifies cell-line identity and does not establish a normal lung epithelial phenotype, developmental progenitor status, or canonical NKX2-1-positive lineage state. This distinction is important because A549 cells have been reported in the literature as NKX2-1/TTF-1-low or negative in several experimental contexts and are often used for exogenous NKX2-1/TTF-1 studies [28,29,30,31]. At the same time, earlier work has reported detectable TTF-1 mRNA and cytoplasmic signal in A549 among lung cancer cell lines, while noting that nuclear TTF-1 staining is uncommon in such lines [32]. The NKX2-1/TTF-1 signal detected in the present A549 culture was therefore treated as a context-specific observation within the authenticated assay background, and the study does not rely on defining A549 as a universal NKX2-1-positive lung-lineage model.

Within this cellular assay framework, the reporter data indicate that the distal and proximal TP63 promoter-associated regions do not behave as equivalent passive DNA fragments (Figure 1, Figure 2 and Figure 3). The distal reporter showed context-dependent effects of FOXA1/2-, SOX9-, and NKX2-1-related motif perturbation, whereas the proximal reporter showed broader positive contributions from several predicted motifs, including FOXA1/2, SOX9, NKX2-1, TP63, and a TATA-box-associated element. These findings indicate that TP63 promoter output is shaped by promoter architecture and motif composition. Because these assays use episomal rabbit promoter fragments outside their native chromatin environment, the results are best interpreted as promoter-fragment responsiveness data that define candidate regulatory intervals for future endogenous testing.

The endogenous ChIP-qPCR assays provide local genomic context for the promoter-reporter findings (Figure 4 and Figure 5). These assays were designed as targeted enrichment analyses at selected TP63 promoter-associated regions and were not intended as comprehensive assessments of genome-wide transcription-factor occupancy. Enrichment of selected transcription factors at human TP63 promoter-associated regions in A549 cells supports the view that the corresponding genomic neighborhoods can be examined within this cellular background. ChIP-qPCR at selected sites is inherently regional and hypothesis-driven, and an independent genomic negative locus outside the tested promoter regions was not included in the present assay design. Previous Nkx2-1 ChIP validation strategies have used promoter-region primers to assess binding and exon-region primers from the same gene as controls [33], and such additional negative-locus controls would further strengthen future validation. Within these boundaries, the ChIP-qPCR data provide supporting local enrichment evidence for the promoter-centered model.

The siRNA perturbation experiments provide supportive endogenous protein-level observations that are consistent with the promoter-reporter findings (Figure 6). Considered alongside the promoter-context-dependent reporter effects and ChIP-qPCR enrichment patterns, these observations suggest that FOXA2 and SOX9-associated regulatory inputs may contribute to TP63 isoform-associated output in the A549 cellular context. Because only one siRNA sequence was used for each target gene and rescue experiments were not performed, these data should be interpreted as supportive perturbation evidence and should not be considered definitive proof of direct causality. Independent siRNAs or rescue experiments would strengthen causal interpretation in future studies [34,35]. In addition, because endogenous TP63 isoform abundance can be influenced by multiple regulatory layers, including transcriptional regulation, post-transcriptional mechanisms, and protein stability, the observed protein-level changes cannot be interpreted as a direct quantitative readout of promoter activity alone. Future studies incorporating isoform-specific transcript quantification and rescue approaches will help further distinguish transcriptional from post-transcriptional regulatory effects. The divergence between proximal reporter activity and endogenous ΔNp63 protein response suggests that intact-cell TP63 isoform abundance may involve additional regulatory layers, including indirect transcriptional network effects, feedback regulation, post-transcriptional control, and protein stability.

The transcription-factor inputs examined here are biologically grounded in epithelial and lung developmental regulatory programs. FOXA factors are required for lung epithelial morphogenesis and differentiation and can cooperate with lung lineage transcriptional networks [9,13,14,15]. NKX2-1 is essential for lung organogenesis and is widely used as a defining marker for lung-lineage progenitors generated from pluripotent stem cells [9,11,12]. SOX2 and SOX9 participate in proximal-distal epithelial patterning, branching morphogenesis, and epithelial progenitor-state regulation [16,17,18,19]. These studies support the biological relevance of testing FOXA-, NKX2-1-, and SOX-related inputs at TP63 promoter-associated regions. The present data further suggest that TP63 promoter fragments contain transcription-factor-responsive architecture whose output depends on promoter context, motif composition, and cellular regulatory background. Future work in primary or stem-cell-derived epithelial systems will be needed to define the physiological developmental roles of these inputs.

The rabbit component provides a practical comparative promoter-template strategy for reporter-based discovery. The resulting reporter activities should therefore be interpreted as promoter-fragment responsiveness and not as direct measurements of endogenous human TP63 promoter regulation. The use of rabbit TP63 promoter regions is justified by practical considerations: relative to mouse or rat, rabbit offers a longer pseudoglandular stage [20,21] and closer phylogenetic [22,23] proximity to human, which together provide a rational and broader developmental window for exploring promoter regulation in a respiratory context. The present study was not designed to compare TP63 isoform expression patterns between rabbit and human systems. Instead, rabbit sequences were used as promoter templates for functional interrogation of candidate regulatory elements in a human epithelial reporter background. These features make rabbit a reasonable comparative source for promoter-fragment discovery. Nevertheless, the sequence alignments in this study showed variable local similarity across the analyzed regions, with mismatches, short gaps, and unresolved sequence intervals in some regions. Therefore, the rabbit promoter reporters are best described as positionally selected promoter-neighborhood fragments containing locally alignable subsegments that can be functionally interrogated in a reporter system.

The main implication of this work is that the two TP63 promoter-associated regions can receive distinct transcription-factor-related inputs in a reporter-based assay. Selected endogenous observations in A549 cells are compatible with this promoter-context-dependent regulatory model. This framework can guide future studies of TP63 isoform-associated promoter logic in more physiological systems such as hiPSC-derived lung progenitors, fetal lung epithelial cells, primary airway basal cells, or organoid models [8,9,10,11,12,19]. In such systems, endogenous promoter mutagenesis, isoform-specific nascent transcript quantification, CUT&RUN/CUT&Tag or ChIP-seq, ATAC-seq, H3K27ac profiling, and perturbation-rescue experiments could be used to test the physiological relevance of the promoter responsiveness observed here.

Several limitations define the boundary of this study. The experiments rely on A549 cells and rabbit promoter-reporter fragments, and the ChIP-qPCR and siRNA assays provide selected supporting evidence but do not exhaustively validate endogenous regulation. These limitations do not negate the promoter-level findings; instead, they indicate that endogenous promoter editing, isoform-specific nascent transcript assays, chromatin profiling, and validation in primary or iPSC-derived lung epithelial systems will be useful next steps for testing the physiological relevance of the promoter-responsiveness model proposed here.

## 5. Conclusions

In summary, this study identifies promoter intervals and predicted transcription-factor-responsive motifs within distal TAp63-associated and proximal ΔNp63-associated TP63 promoter-reporter fragments. Serial deletion and motif perturbation showed that FOXA1/2-, NKX2-1-, SOX9-, and TP63-related inputs influence reporter output in a promoter-context-dependent manner. Endogenous ChIP-qPCR and FOXA2/SOX9 perturbation in STR-authenticated A549 cells provided supporting local and protein-level observations consistent with this promoter-level model. These findings define a promoter-responsiveness framework for investigating TP63 dual-promoter regulatory logic and provide a basis for future validation in primary or stem-cell-derived epithelial systems.

## Figures and Tables

**Table 1:** Primers used for creating full-length and deletion constructs of rabbit TP63 gene promoter.

Subject		Product	Primer Sequence (5′–3′)
high-fidelity PCR for full-length promoter	distal promoter	D	F: AAAAAAGGTACCCATGCAATCAGGTCATGTGCTAGGCTT R: AAAAAAGCTAGCTGTATAGTAGCCACTCTTCTTGTTGGC
	proximal promoter	P	F: AAAAAAGGTACCTTACCAGCCAGCTATACCACCACACC R: AAAAAAGCTAGCATTTTGCCAGCCCCCCACCCCCTCT
high-fidelity PCR for deletion constructs	distal promoter deletion constructs	D1	F: AAAAAAGGTACCAATTGATTGTTTTGAAATTTTATAGCCTAGGATG
		D2	F: AAAAAAGGTACCTCCTTTCCATCCCCGCTGAAGCA
		D3	F: AAAAAAGGTACCCCAACATAGGCTTTCAGTCA
		D4	F: AAAAAAGGTACCTGTTTGAAGCAGGATATTTAAGCAT
		D5	F: AAAAAAGGTACCAGAAACATTTTAACCCCAGAAGC
		D6	F: AAAAAAGGTACCTACAAAAGTCCAGGCTGCTGA
	proximal promoter deletion constructs	P1	F: AAAAAAGGTACCAGTGTCTATGCAATTCAGCTGCTTC
		P2	F: AAAAAAGGTACCTACCTGCAAGGAAACATCTATCTGCTTT
		P3	F: AAAAAAGGTACCTCAAATGGCTTGTTTCCCAG
		P4	F: AAAAAAGGTACCCTGAAATGCCTTCTCTAAGTCTTGGTG
		P5	F: AAAAAAGGTACCATTCTAACTACTTAATGAGATGGGAG
		P6	F: AAAAAAGGTACCGAAGAGTCCCGCCCCTCATGC

Notes: Restriction sites underlined are KpnI (GGTACC) and NheI (GCTAGC) in expression vector pGL4.17.

**Table 2:** Primers used for creating mutant constructs of rabbit TP63 gene promoter.

Subject	Product	TFs	Mutated Sequence	Primer Sequence (5′–3′)
high-fidelity PCR for distal promoter mutant constructs	P41S9	SOX9	CTATAGATT→×	F: CCTGAAAAGAAGTCTCACAACTTTGACATCTGGGA R: GTGAGACTTCTTTTCAGGTAAATTTGGTATTTCC
	P41F12	FOXA1/FOXA2	ATGTGAACATGC→×	F: GCATGTTAGAGACACTGGGAACACACATTTCTT R: CCAGTGTCTCTAACATGCTTCCATTCATTTTTAATCACAGGA
	P42S2	SOX2	GAACAATTTTA→×	F: CTCAGTTTCATTCCAAAGGAGGTCAAGTCAAAG R: CTTTGGAATGAAACTGAGAAATTCCAAAACTTAATATCAGGAAA
	P42N	NKX2-1	CATTTGAA→×	F: GAAGCAGGATATTTAAGTAAAGTTTATTTTATTTGGGGCAAGC R: ACTTAAATATCCTGCTTCAAACACACACCCACA
high-fidelity PCR for proximal promoter mutant constructs	P5S9	SOX9	CAATTGTAT→×	F: CAGCTGCTTAATACAGAGATGAGCAAATCGAGGG R: CTCTGTATTAAGCAGCTGAATTGCATAGACACT
	P5F2	FOXA2	ATATAGACATGT→×	F: TAGAGACTGAGCTCTAGCTTTCAAGGTACAAAT R: GCTAGAGCTCAGTCTCTATACAATAAGAATAAAAATAAAAAACTTCATTT
	P5F1	FOXA1	CGTTTGCTCTGG→×	F: CAGTCGTGCTAACAGAGTATTCCTTCTGTGTGTGACTCACTC R: ACTCTGTTAGCACGACTGCCCAGAACGGCATTA
	P5N	NKX2-1	AGCACTGGAGCCA→×	F: GTCTGATTCTGTGATCCAAAAATACACTTTAAAAAGCAC R: TGGATCACAGAATCAGACTAAGTCTGCAGCCCA
	P51S9	SOX9	ATTGCTCCT→×	F: CCACTTAAACTGGCAGTGAGTGCAGTGTTTAGA R: CACTGCCAGTTTAAGTGGAAAGCAGATAGATGTTTCCTTG
	P51F2	FOXA2	TGTTTACATATGT→×	F: GGGTGGAAGATCTAACTGAATTATGAATACTTAGTAGTAATGAGA R: CAGTTAGATCTTCCACCCTTACTCAGGAAGCAA
	P51F1	FOXA1	ATGTAAATATAT→×	F: GAATGTTCTTATGTATTTTTTTAAAGTACAAACTAATTTGTTTCTCCT R: AAATACATAAGAACATTCAGACAGAATGTGGAC
	P51TAP	TP63	CATGCTCTGAAAAGTCAA→×	F: AAAGTGGTTTATACTGGCATTGCTTGGTGTGAA R: GCCAGTATAAACCACTTTTAGTGACAATTTAAGCTCTTGGTCC
	P54N1	NKX2-1	TTGGAGTGG→×	F: TCACTCCAGAGGAGTCCAGGTGGAAGTTGATGG R: TGGACTCCTCTGGAGTGAGGCCTCTCCCATCTC
	P54TF	TATA-binding protein	TATATTA→×	F: GCCTATAGTTGAGTAGGGAACCTTAAGTTATGTACAGAGAGA R: CCTACTCAACTTAGGCATGAGGGGCGGGACT

**Table 3:** Primers used for ChIP-qPCR targeting TFBSs in the promoter region of the human TP63 gene.

Subject	TFBSs	TFs	Product Length	Tm	Primer Sequence (5′–3′)
Human TAp63 promoter	P41F12	FOXA2	167 bp	55 °C	F: GGGATGTCACCAGTTTACAG R: ATGTTGGCTTTGTGGATCTG
	P42N	NKX2-1	171 bp	54.5 °C	F: GGTGTGTGTTTGAAGCAGGA R: TCTCAAATAGCAAAGACAGGGA
	P41S9	SOX9	186 bp	56 °C	F: TTAAATAGCTTCTCCTTTCCATCC R: CATTCATTCTTTAATCACAGGACAC
Human ΔNp63 promoter	P5F2	FOXA2	150 bp	54.5 °C	F: CCTGCTGGGAATCTTTTGAC R: CGGCATTATTAGATTCTTTTACACA
	P51F2	FOXA2	154 bp	54.5 °C	F: TCATTTATAGTTATCTTGGCCAGT R: TCGAAGGTTGTTATTTGATTCCT
	P5N	NKX2-1	150 bp	53 °C	F: CCTAGTAGGCTATATAAAATCCAGT R: GTATGCTCTAGTCTAGTGATTCAAA
	P54N1	NKX2-1	212 bp	55 °C	F: AGATTGGTGATAAGGAATTCTAACT R: CCTAAGAAGATAACAGCACTCG
	P5S9	SOX9	187 bp	53.5 °C	F: ATTGTAGGCATATCCTGGAACA R: AGTGAAAGTATTACTCACAATTCTC
	P51S9	SOX9	184 bp	55 °C	F: GCACTTCTGATGATTTGCAG R: TGCCTACAAGGCAAACAAAT
	P51TAP	TP63	185 bp	53.5 °C	F: AAATGGCTTGTTTCCCAGAA R: ATTTTCAATTTATTTTCCCCAGAGA

## Data Availability

All public transcriptomic datasets analyzed in this study are available from the Gene Expression Omnibus under the accession numbers listed in Supplementary Table S2. The original data generated in this study, including promoter reporter assays, ChIP-qPCR data, immunoblot densitometry, uncropped gel/blot images, and related analyses, are included in the article and Supplementary Materials. Additional raw data supporting the findings are available from the corresponding author upon reasonable request.

## Supplementary Materials

The following supporting information can be downloaded at: https://www.mdpi.com/article/10.3390/cimb48090882/s1.
