# Supplementary material for: Functional Dissection of TP63 Dual-Promoter Regulatory Elements Reveals Context-Dependent Responsiveness to FOXA2, NKX2–1, SOX9, and TP63-Related Inputs
Source: Curr Issues Mol Biol. 2026 Aug 30;48(9):882. doi: 10.3390/cimb48090882 (PMC13605174; doi:10.3390/cimb48090882)
Supplement: Supplementary file 1 [file cimb-48-00882-s001.zip › 02_Supplementary_File_S1/Supplementary File S1.pdf]

A549 [A-549] Cell STR Authentication Report

English translation of the original Chinese certificate

1. Sample Processing and Testing Methods

An appropriate amount of A549 [A-549] cells (No. PC-H2025022738, 1×10^6) was collected. Genomic DNA was extracted using the TIANamp Genomic DNA Kit. Twenty STR loci and the sex-determination locus were amplified using the Microreader™21 ID System. PCR products were detected using a GenReader 7010 Genetic Analyzer. The test results were analyzed using GeneMapper Software 6 (Applied Biosystems) and compared with the ExPASy database.

2. Test Results

The negative and positive controls in the experiment produced the expected results.

The genotyping results for the STR loci and the Amelogenin locus of the A549 [A-549] cell line are shown in Appendix Table 1. The corresponding electropherogram is shown in Appendix Figure 1.

3. Analysis

The electropherogram obtained after amplification of genomic DNA from the A549 [A-549] cell line showed clear peaks, and the genotyping results were satisfactory.

4. Test Conclusion

1. STR profiling of DNA from the A549 [A-549] cell line showed no evidence of cross-contamination with other human cell lines.

2. Comparison of the DNA profile with the cell-line database identified a cell line with a 100.00% matching profile, corresponding to A-549.

Website: www.procell.com.cn
Tel: 400-999-2100
Email: techsupport@procell.com.cn
Address: Building C4, Phase III, Biopharmaceutical Industrial Park, No. 858 Gaoxin Avenue, Wuhan, Hubei, China

Appendix Table 1. Genotyping results for the STR loci and Amelogenin locus of the A549 [A-549] cell line.

STR Locus	Sample Name: PC-H2025022738	Database Name: A-549
Amelogenin	X, Y	X, Y
CSF1PO	10,12	10,12
D2S1338	24	24
D3S1358	16	16
D5S818	11	11
D7S820	8,11	8,11
D8S1179	13,14	13,14
D13S317	11	11
D16S539	11,12	11,12
D18S51	14,17	14,17
D19S433	13	13
D21S11	29	29
FGA	23	23
Penta D	9	9
Penta E	7,11	7,11
TH01	8,9.3	8,9.3
TPOX	8,11	8,11
vWA	14	14
D6S1043	11,13	
D12S391	18	18
D2S441	10,13	10,13

ExPASy database match: 100.00%; number of matched loci: 19 (https://www.cellosaurus.org/index.html)

Notes:

1. According to the cell STR authentication criteria established by the International Cell Line Authentication Committee (ICLAC), a cell-line match of ≥80% indicates relatedness, i.e., derivation from a common ancestral cell line. A match between 55% and 80% requires further verification of relatedness; a match below 55% indicates that the two cell lines are not related.

2. In the electropherogram, valid peaks represent true PCR products; stutter peaks and nonspecific bands are excluded from the calculation.

3. By default, STR database comparison uses ExPASy. Data sources include cell repositories such as ATCC, DSMZ, and JCRB, as well as published literature and data records. Database entry: https://www.cellosaurus.org/index.html.

Appendix Figure 1. Genotyping profile of the STR loci and Amelogenin locus of A549 [A-549] cells (No. PC-H2025022738).

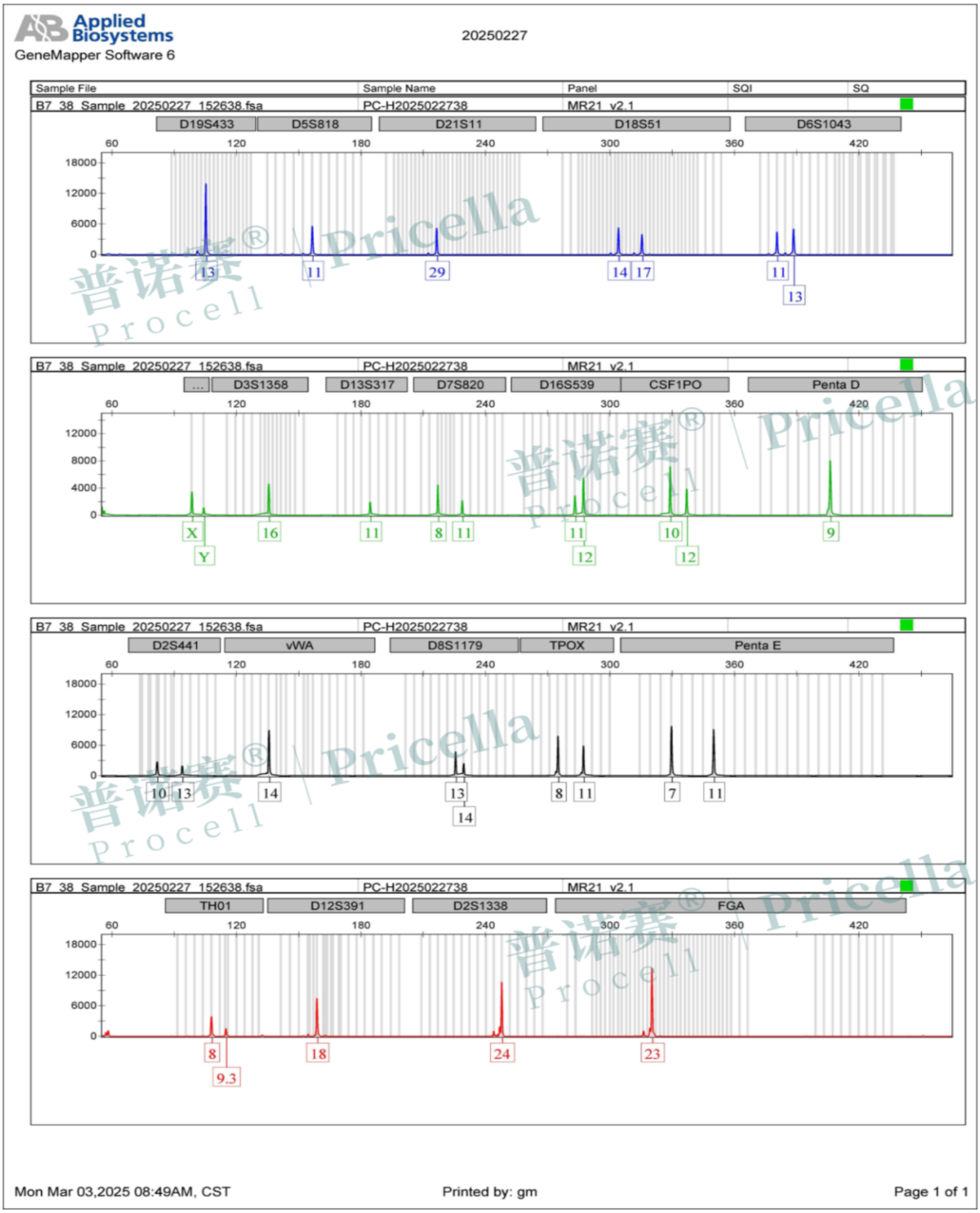

The GeneMapper electropherogram above is reproduced from the original authentication certificate; all allele calls and numerical results are unchanged.
