# Supplementary material for: Functional Dissection of TP63 Dual-Promoter Regulatory Elements Reveals Context-Dependent Responsiveness to FOXA2, NKX2–1, SOX9, and TP63-Related Inputs
Source: Curr Issues Mol Biol. 2026 Aug 30;48(9):882. doi: 10.3390/cimb48090882 (PMC13605174; doi:10.3390/cimb48090882)

Supplementary Table Legend

Supplementary Table S1. Predicted transcription factor binding motifs within candidate regulatory segments of the rabbit TP63 distal and proximal promoter reporters.

Predicted TFBSs were identified using JASPAR in the deletion-defined candidate regions of the rabbit TP63 distal promoter (Da and Db) and proximal promoter (Pa, Pb, and Pc). The table lists the transcription factor, site label, promoter region, predicted motif sequence, and related annotation used to guide site-directed mutagenesis and downstream reporter assays.

Supplementary Table S2. Sample metadata used for the gene expression contextualization.

The table lists the GEO sample accessions and source filenames for the datasets included in the comparative gene expression analysis shown in Figure 7. Samples were grouped as A549 cells, hiPSC-derived developmental reference states, and primary respiratory epithelial reference populations. This table is intended to document sample provenance for the PCA, heatmap, and related downstream analyses.

Supplementary Table S3. Genes within the top 20% of absolute loadings for PC1 and PC2 derived from the PCA.

The table lists the genes with the largest absolute loadings on PC1 and PC2 from the PCA performed on the full filtered gene expression datasets used to position A549 relative to developmental and respiratory epithelial reference samples. For each gene, the signed loading, absolute loading, loading direction, and rank within the respective principal component are provided. These contributor-gene sets were used for the heatmap visualization shown in Figure 7B and for the downstream Gene Ontology analysis summarized in Supplementary Table S4.

Supplementary Table S4. Gene Ontology biological process analysis of the top 20% absolute loading genes for PC1 and PC2.

The table summarizes the Gene Ontology biological process enrichment results for the PC1 and PC2 contributor-gene sets listed in Supplementary Table S3, which were selected based on absolute loading values from the PCA shown in Figure 7A. The full enrichment output is provided in the main GO result sheets, and the additional sheet "Terms_plotted_in_Fig7C" lists the lung-related terms selected from the enrichment results for visualization in Figure 7C.

Supplementary File Legend

Supplementary File S1. Supplier-provided STR authentication report for the A549 cells used in this study.

The file contains the supplier-provided short tandem repeat (STR) authentication report for the A549 cell line used in the experiments. The report was used to verify cell-line identity.

Supplementary Figure Legend

Supplementary Figure S1. Full agarose gel images of the reporter constructs used in this study after double-enzyme digestion.

Full-length and truncated distal and proximal rabbit TP63 promoter fragments cloned into the pGL4.17 vector were verified by agarose gel electrophoresis following double digestion.

Supplementary Figures S2-S4. Public chromatin-context maps across the human TP63 locus in A549 cells.

Public A549 Hi-C interaction maps were visualized in the 3D Genome Browser and displayed together with promoter-like signatures (PLS), proximal enhancer-like signatures (pELS), distal enhancer-like signatures (dELS), CTCF-associated elements, and RefSeq gene models. Supplementary Figure S2 shows a broad overview of the TP63 locus, whereas Supplementary Figure S3 and Supplementary Figure S4 show enlarged views centered on the

TAp63 and ΔNp63 promoter neighborhoods, respectively.

Supplementary Figures S5-S6. Alignment of TP63 promoter regions analyzed in Figures 4-5 across human, mouse, and rabbit.

Supplementary Figure S5 shows the distal promoter regions corresponding to P41F12, P42N, and P41S9. Supplementary Figure S6 shows the proximal promoter regions corresponding to P5F2, P51F2, P5N, P54N1, P5S9, P51S9, and P51TAP. The alignments show variable local sequence similarity among the three species and are provided as sequence-context information for the analyzed regions.

Supplementary Figure S7. Full agarose gel images corresponding to the endogenous ChIP-qPCR amplicons shown in Figures 4-5.

For each analyzed site, the complete gel image includes the molecular marker, input, IgG, and antibody-immunoprecipitated lanes. Red boxes indicate the regions used for the representative gel panels shown in the main figures.

Supplementary Figure S8. Full-membrane immunoblot images corresponding to Figure 6.

Full-membrane immunoblot images showing FOXA2, SOX9, TAp63, ΔNp63, and GAPDH signals in si-Ctrl-, si-FOXA2-, and si-SOX9-treated A549 cells. Red boxes indicate the cropped regions used for the representative immunoblot panels shown in Figure 6. For each target blot, all lanes used for comparison were derived from the same membrane and acquired under identical exposure settings. GAPDH was detected on the same membrane as the corresponding target protein and used for lane-wise normalization. The ΔNp63/TAp63 ratio in Figure 6E was calculated from GAPDH-normalized ΔNp63 and TAp63 densitometry values from matched biological replicates. Molecular weight markers are shown to indicate the approximate migration positions of the target bands.

Supplementary Figure S9. Detection of selected epithelial- and transcription-factor-associated proteins in A549 cells by immunoblotting and immunofluorescence.

(A) Immunoblot analysis was performed to examine detectable FOXA2, NKX2-1/TTF-1, SOX9, TAp63, and ΔNp63 signals in the A549 cells used in this study. (B) Band intensities

were normalized to GAPDH and presented as relative normalized protein abundance. (C) Immunofluorescence staining was further performed to examine TP63 isoform-associated signals in A549 cells. Nuclei were counterstained with DAPI (blue), TAp63 is shown in red, and ΔNp63 is shown in green. (D) Immunofluorescence intensity was quantified as the mean fluorescence intensity of the target signal normalized to the mean DAPI intensity in the corresponding field. Scale bar, 100 μm.

Supplementary Figure S10. Full-membrane immunoblot images corresponding to Supplementary Figure S9.

Full-membrane immunoblot images showing antibody-detected FOXA2 (A), NKX2-1/TTF-1 (B), SOX9 (C), TAp63 (D), ΔNp63 (E), and GAPDH signals in the A549 cells used in this study. Red boxes indicate the cropped regions used for the representative immunoblot panels shown in Supplementary Figure S9. For each membrane, the target protein and GAPDH were detected on the same membrane using species-distinct primary antibodies, followed by simultaneous incubation with HRP-conjugated anti-rabbit and anti-mouse secondary antibodies and chemiluminescent detection. GAPDH was used as the loading control for normalization. Molecular weight markers are shown to indicate the approximate migration positions of the target bands.

Supplementary Figure S11. Enlarged Sanger sequencing chromatograms confirming site-directed mutations of rabbit TP63 promoter motifs.

Enlarged Sanger sequencing chromatograms corresponding to the representative sequencing traces shown in Figure 2A and Figure 3A. (A) Distal-promoter chromatograms corresponding to Figure 2A. (B) Proximal-promoter chromatograms corresponding to Figure 3A. The expanded views facilitate visualization of the introduced nucleotide substitutions in the indicated rabbit TP63 promoter motif mutants.

Supplementary Figure

Supplementary Figure S1. Full agarose gel images of the reporter constructs used in this study after double-enzyme digestion.

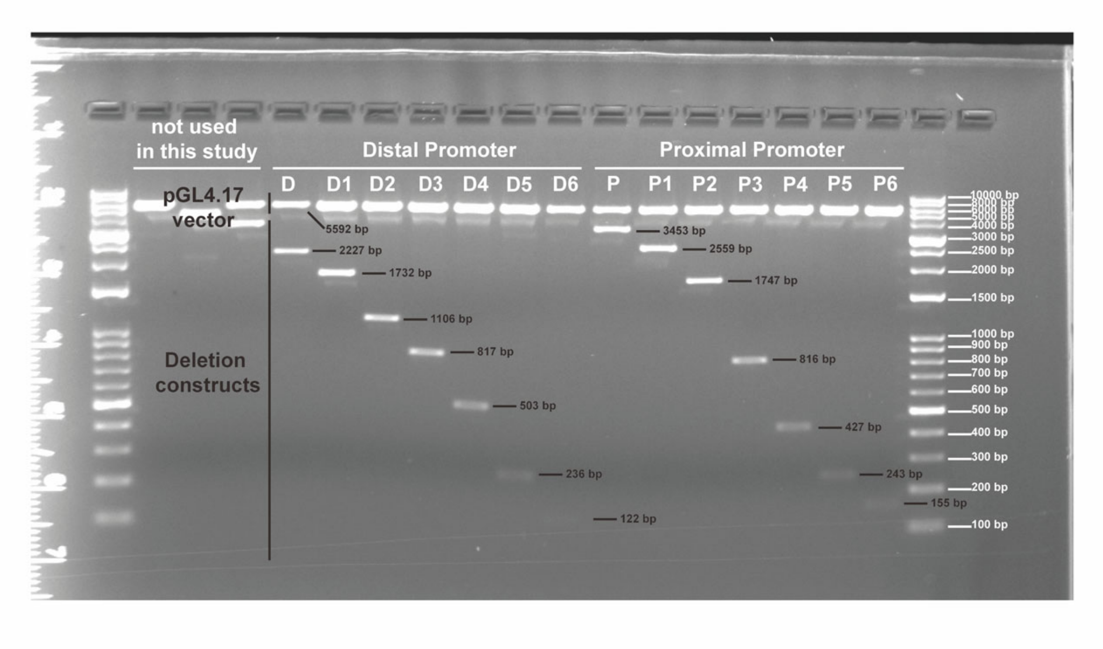

Supplementary Figures S2-S4. Public chromatin-context maps across the human TP63 locus in A549 cells.

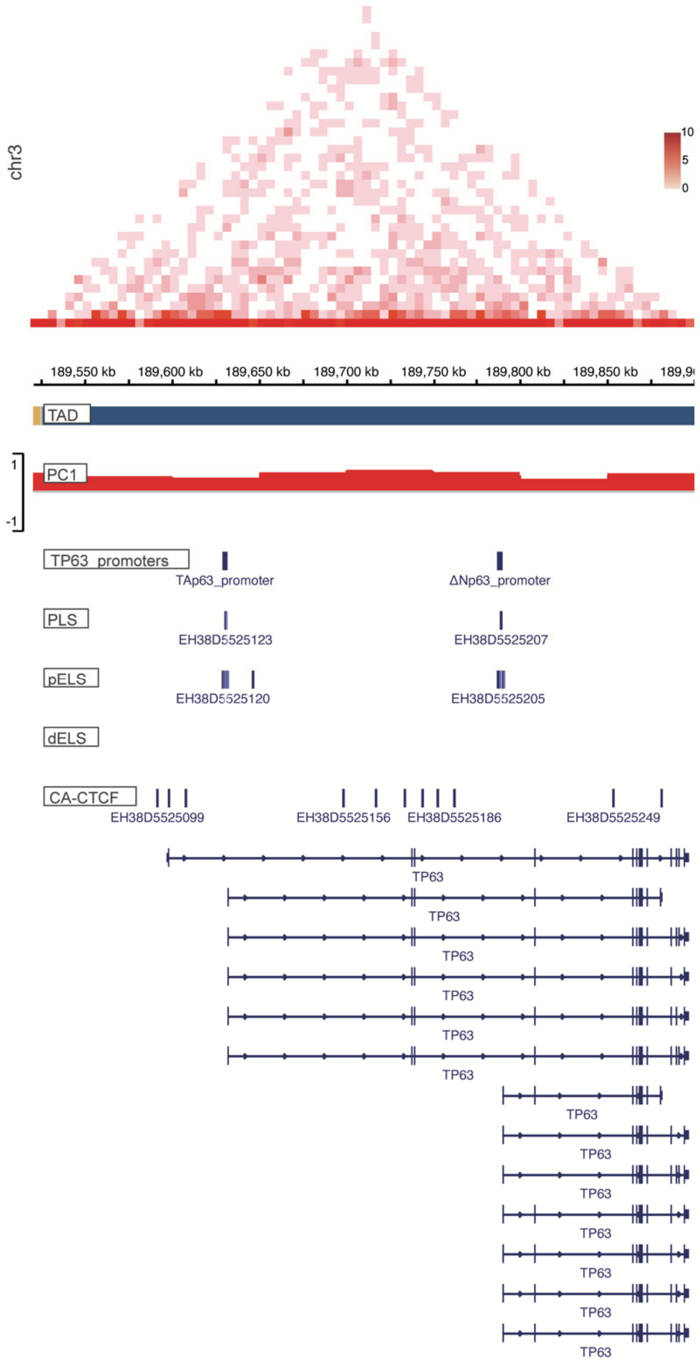

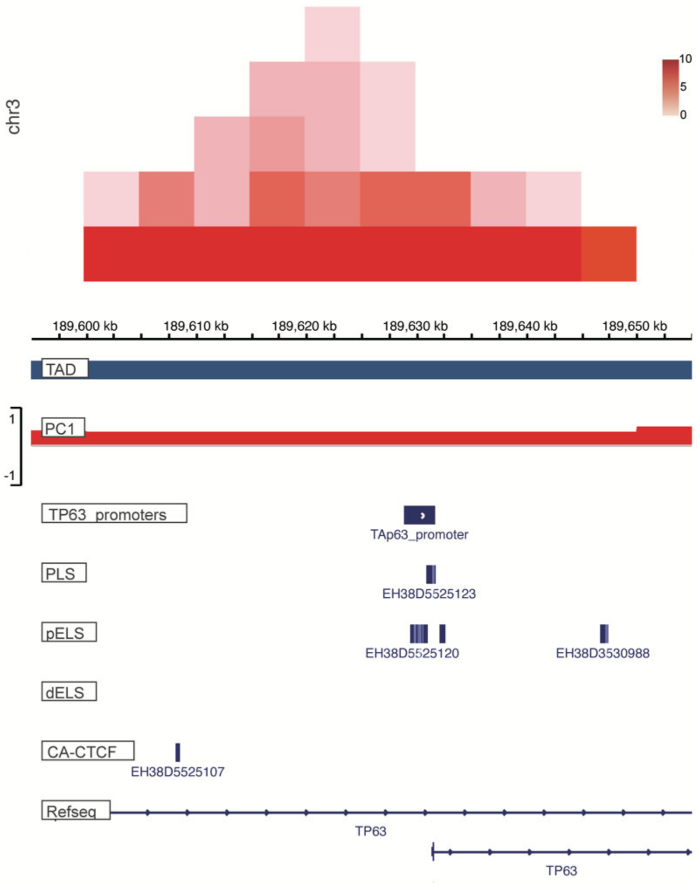

chr3
10
5
0
189,600 kb
189,610 kb
189,620 kb
189,630 kb
189,640 kb
189,650 kb
TAD
1
-1
PC1
TP63_promoters
TAp63_promoter
PLS
EH38D5525123
pELS
EH38D5525120
EH38D3530988
dELS
CA-CTCF
EH38D5525107
Refseq
TP63
TP63

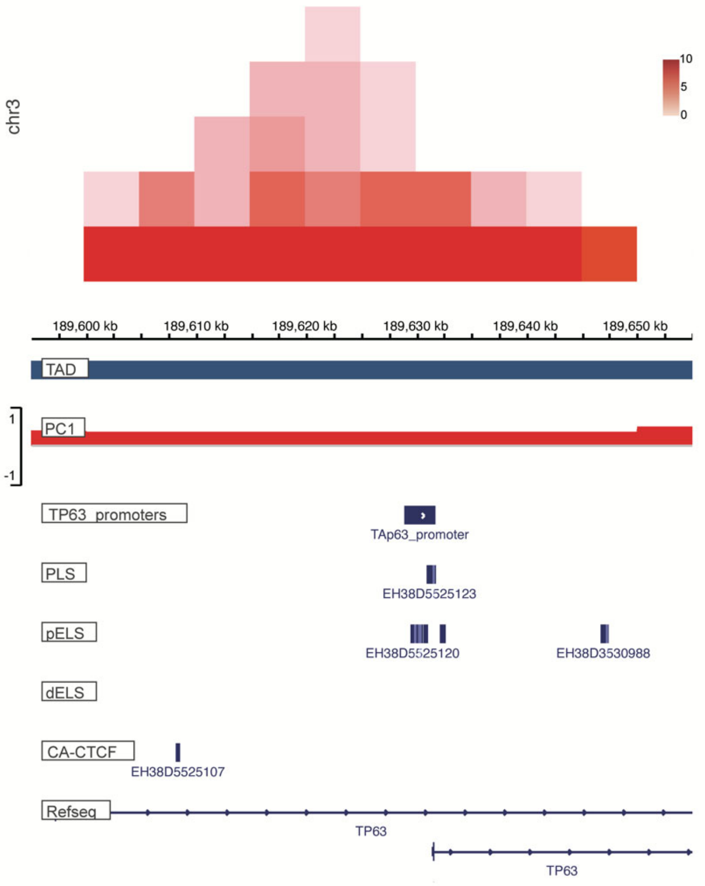

chr3
10
5
0
189,600 kb
189,610 kb
189,620 kb
189,630 kb
189,640 kb
189,650 kb
TAD
1
PC1
-1
TP63_promoters
TAp63_promoter
PLS
EH38D5525123
pELS
EH38D5525120
EH38D3530988
dELS
CA-CTCF
EH38D5525107
Refseq
TP63
TP63

Supplementary Figures S5-S6. Alignment of TP63 promoter regions analyzed in Figures 4-5 across human, mouse, and rabbit.

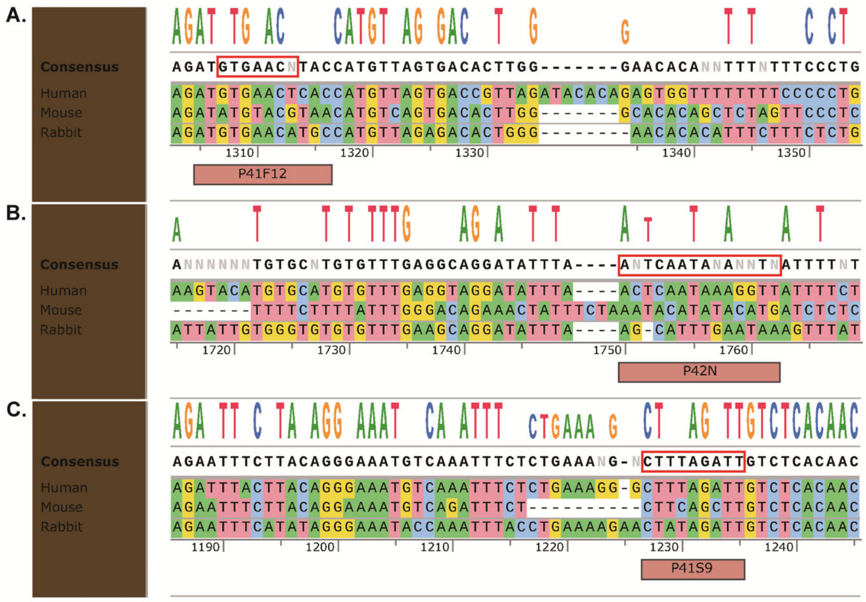

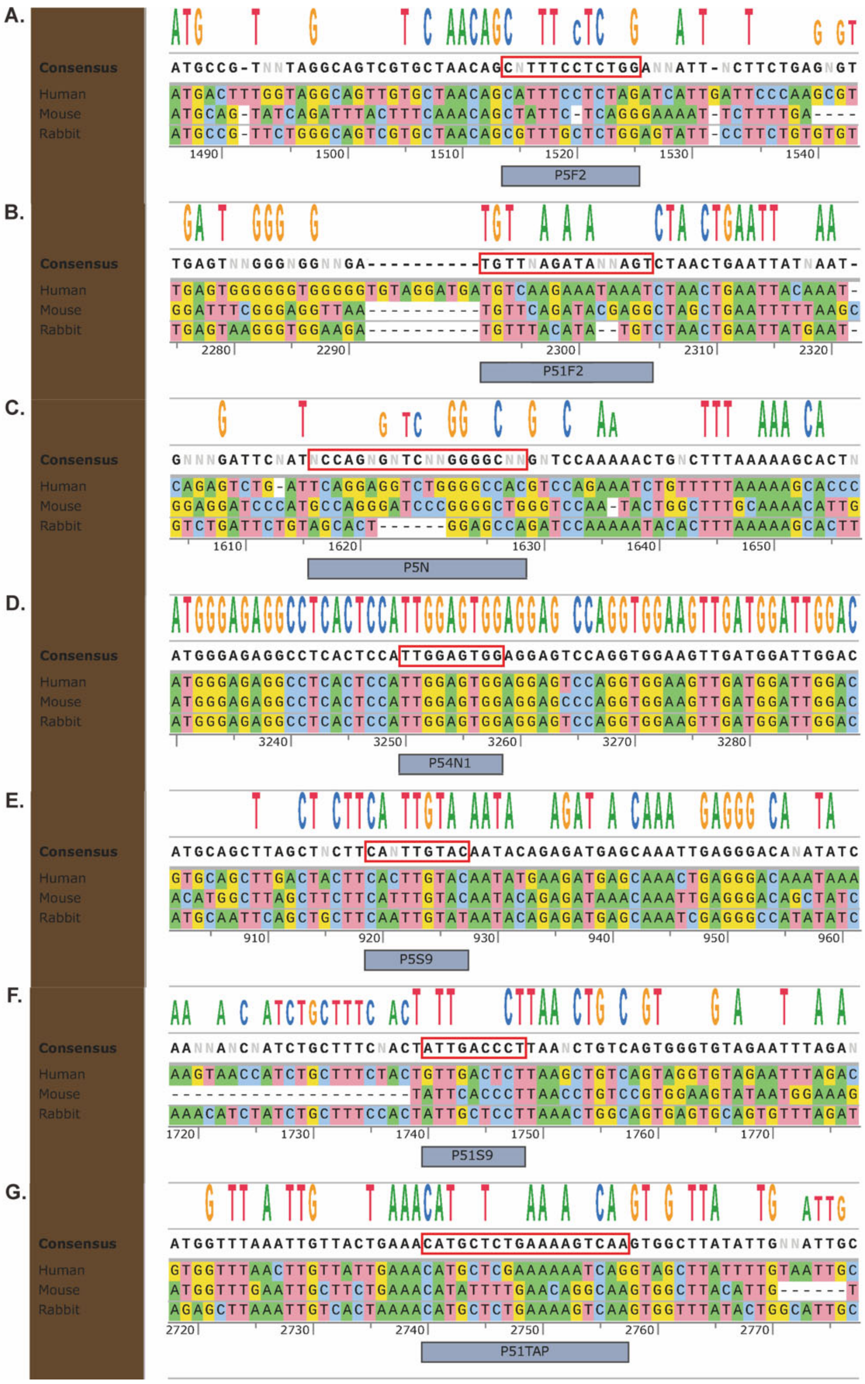

A.
Consensus
Human
Mouse
Rabbit
P5F2
B.
P51F2
C.
P5N
D.
P54N1
E.
P5S9
F.
P51S9
G.
P51TAP

Supplementary Figure S7. Full agarose gel images corresponding to the endogenous ChIP-qPCR amplicons shown in Figures 4-5.

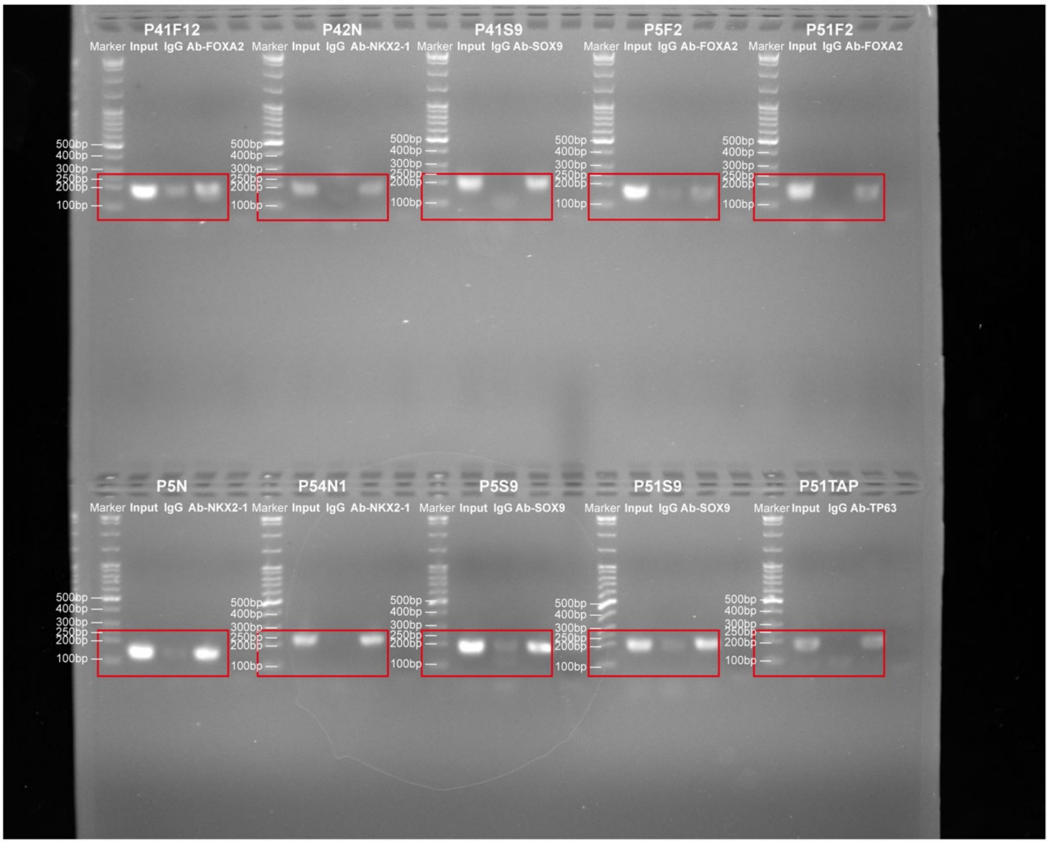

Supplementary Figure S8. Full-membrane immunoblot images corresponding to Figure 6.

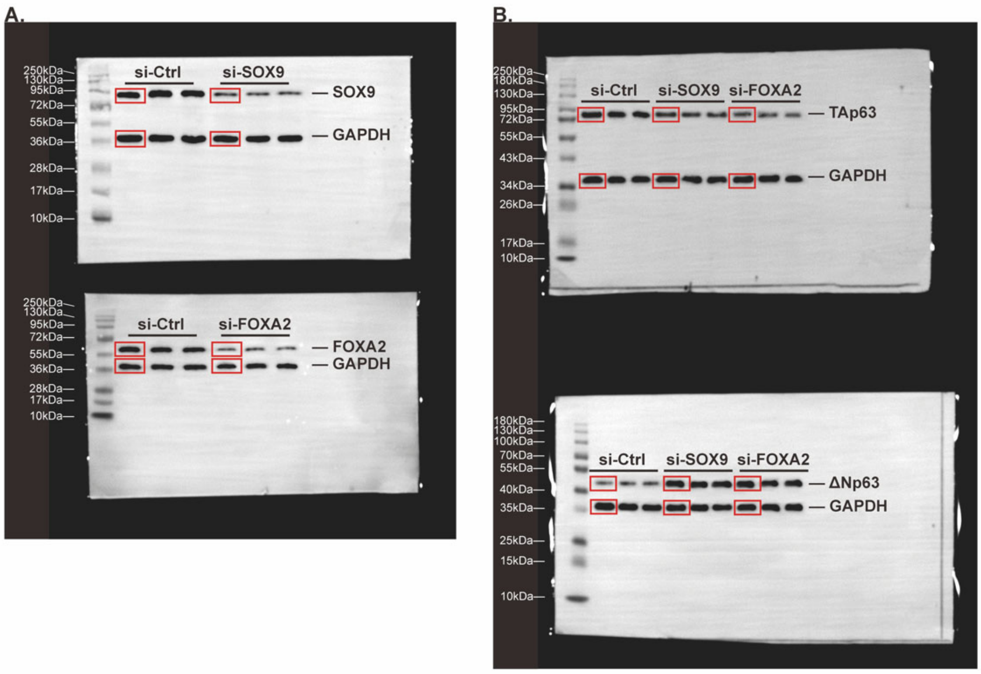

Supplementary Figure S9. Detection of selected epithelial- and transcription-factor-associated proteins in A549 cells by immunoblotting and immunofluorescence.

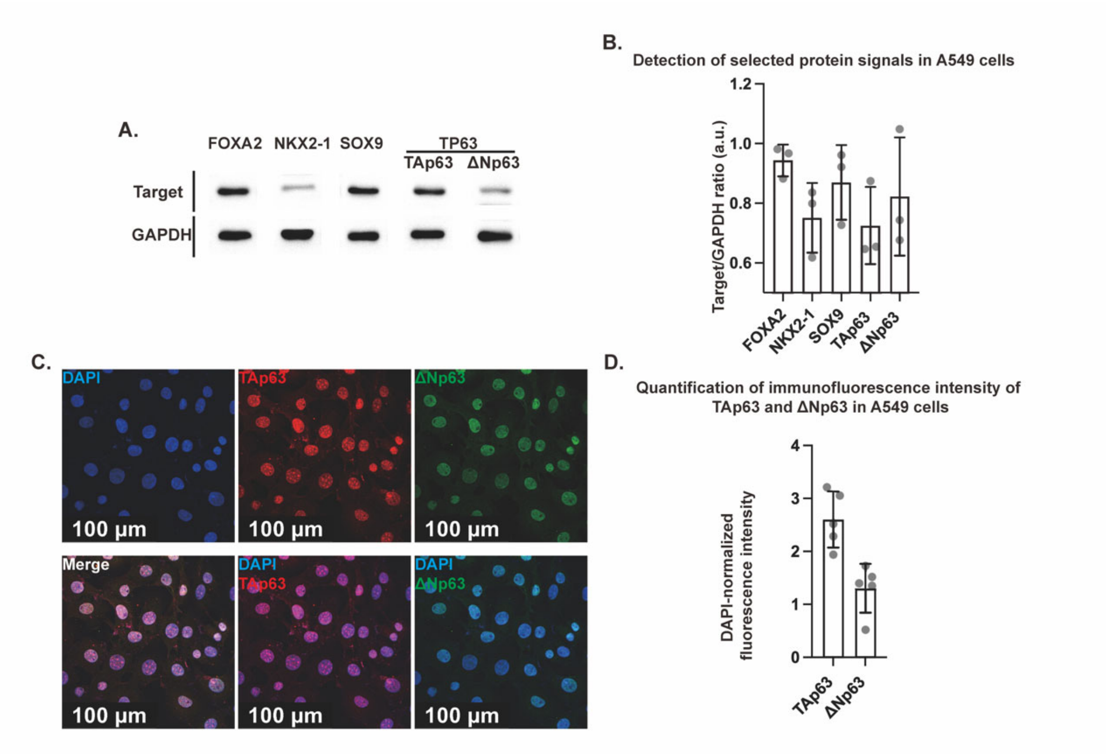

Supplementary Figure S10. Full-membrane immunoblot images corresponding to Supplementary Figure S9.

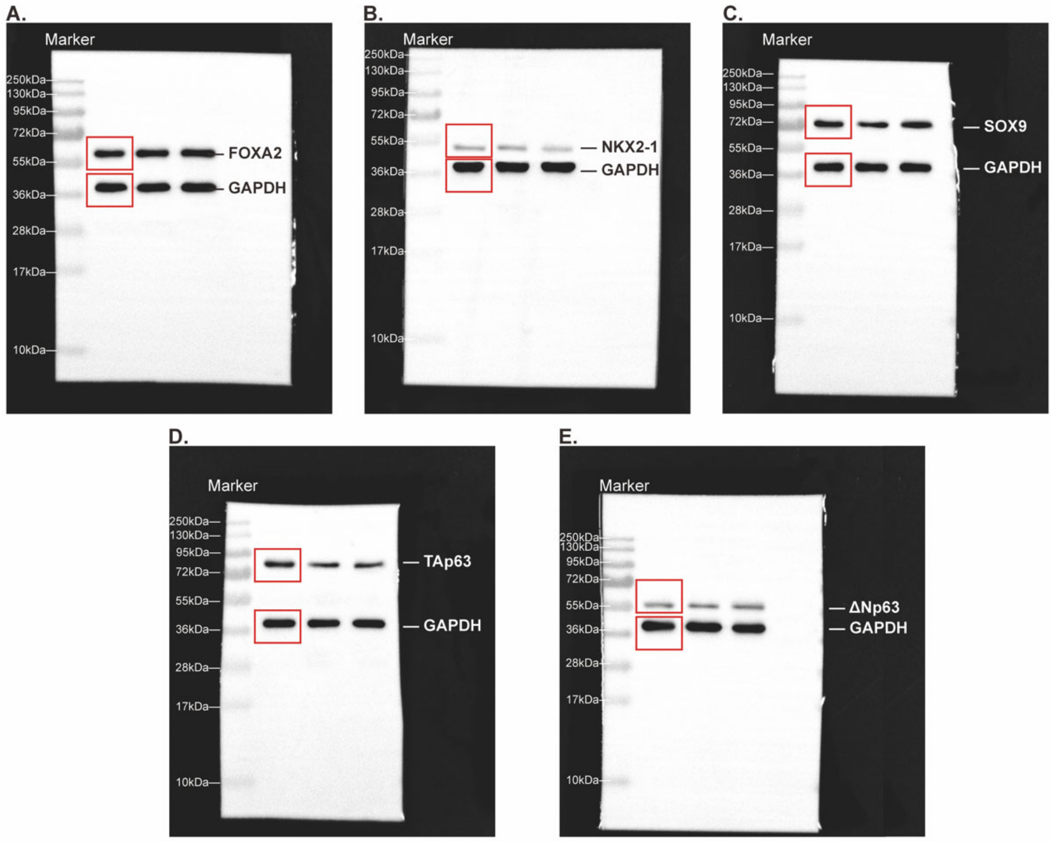

Supplementary Figure S11. Enlarged Sanger sequencing chromatograms confirming site-directed mutations of rabbit TP63 promoter motifs.

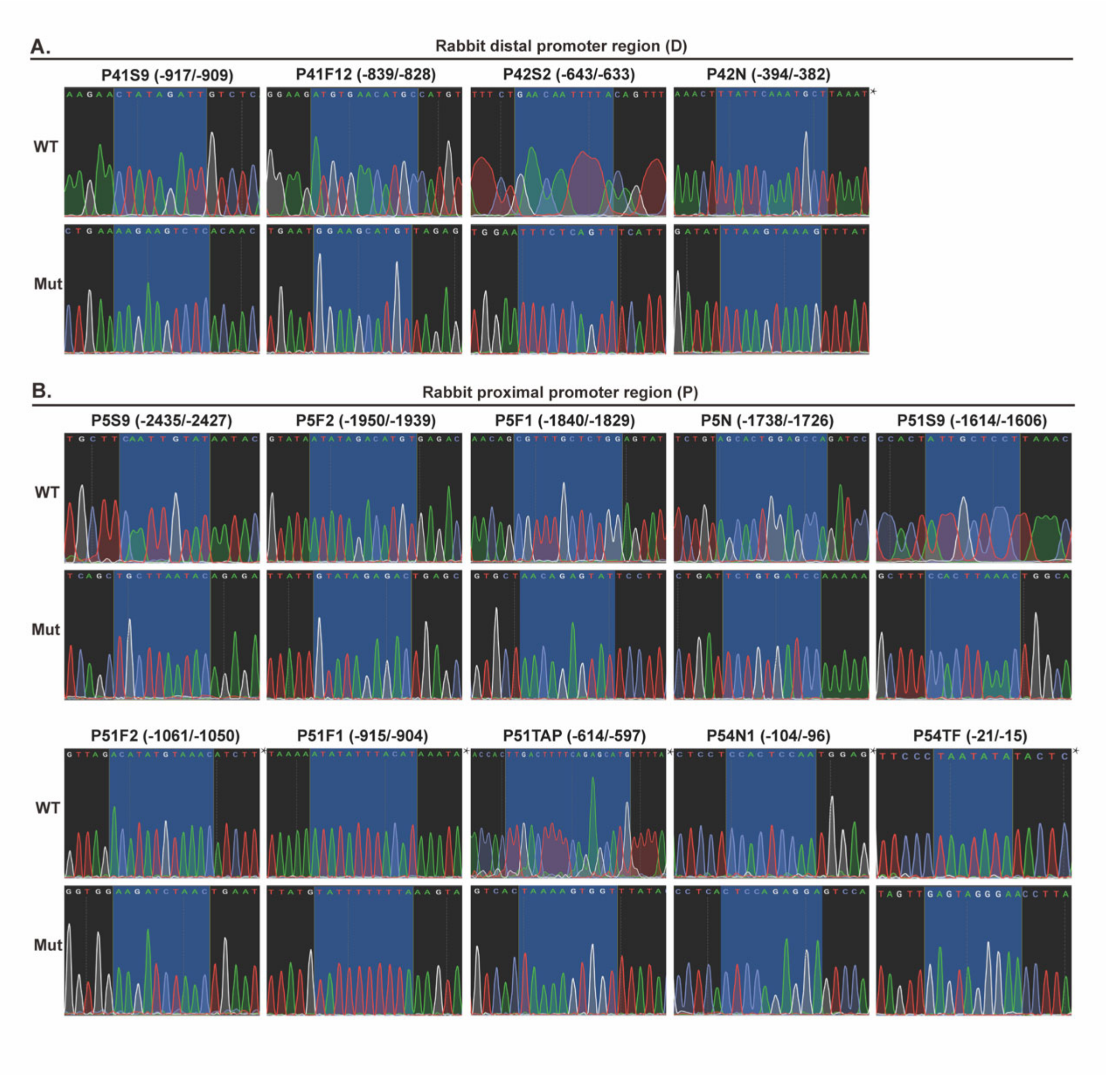

Supplement: Supplementary file 1 [file cimb-48-00882-s001.zip › cimb-4496163-supplementary.pdf]
